# Supplementary material for: Characterization of PIK3CA and PIK3R1 somatic mutations in Chinese breast cancer patients
Source: Nat Commun. 2018 Apr 10;9:1357. doi: 10.1038/s41467-018-03867-9 (PMC5893593; doi:10.1038/s41467-018-03867-9)
Supplement: Supplementary file 1 — Supplementary Information [file 41467_2018_3867_MOESM1_ESM.pdf]

Supplementary Information

Characterization of *PIK3CA* and *PIK3R1* somatic mutations in Chinese breast cancer patients

Chen et al.

Supplementary Figure 1

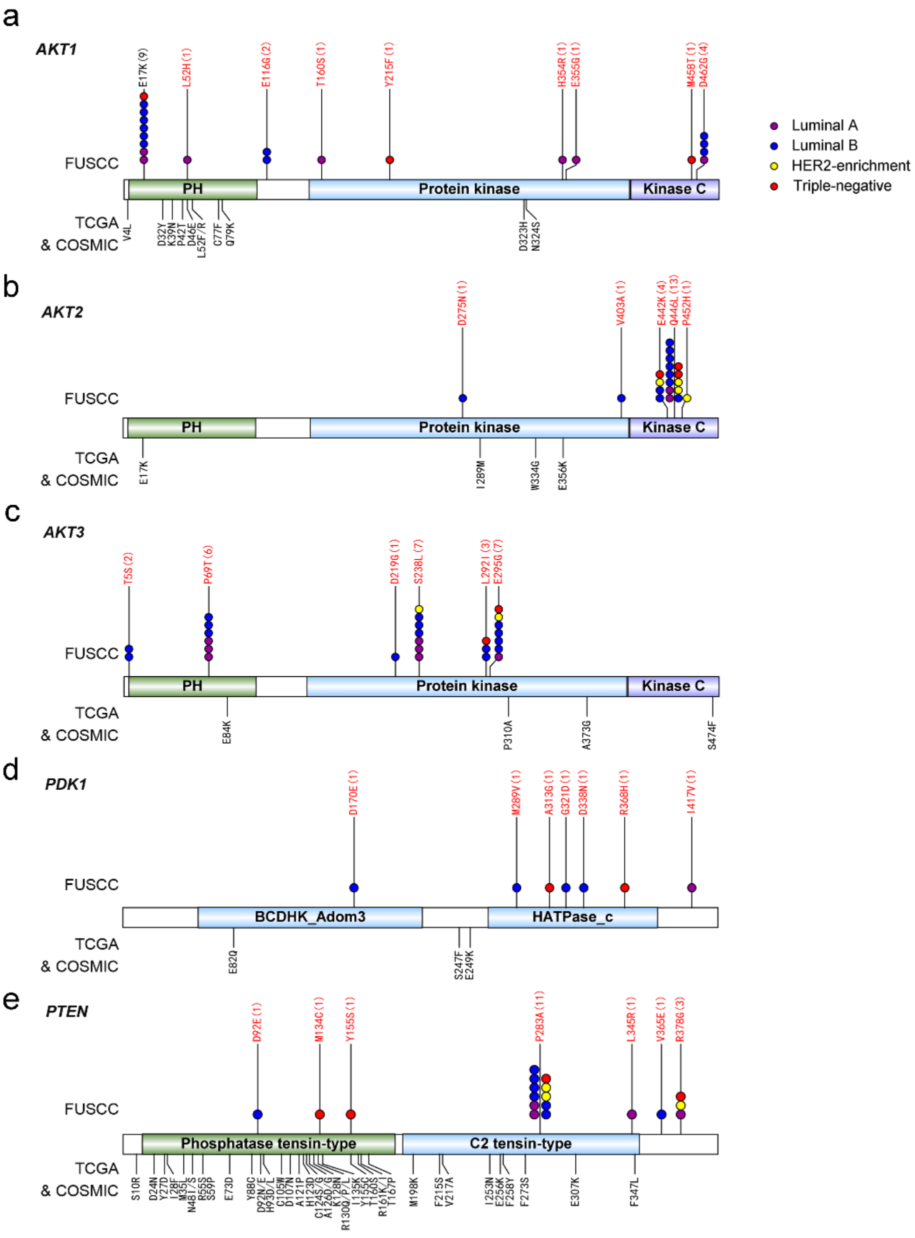

Supplementary Figure 1. The positional distribution of somatic missense mutations in **(a)** *AKT1,* **(b)** *AKT2*, **(c)** *AKT3*, **(d)** *PDK1* and **(e)** *PTEN* from FUSCC dataset (up) and TCGA & COSMIC datasets (down).

Supplementary Figure 2

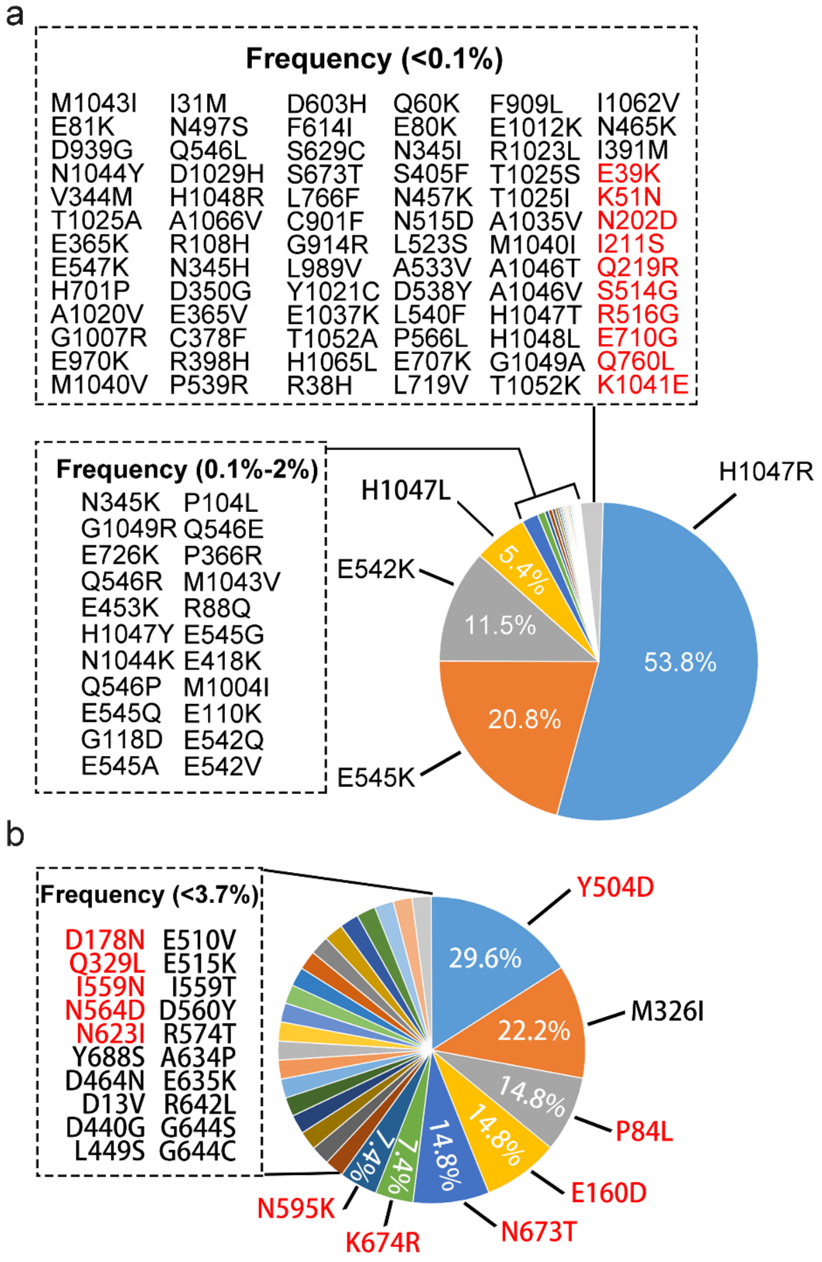

Supplementary Figure 2. *PIK3CA* and *PIK3R1* mutation profiles. **(a)** *PIK3CA* mutations profile and corresponding frequencies, including 94 representative mutations in COSMIC database whose frequencies ranged from 0.02% to 54% in breast cancer. The 10 novel mutations (in red) detected in our cohort were added to the mutations profile. **(b)** *PIK3R1* mutations profile and corresponding frequencies, subsuming 16 missense mutations in COSMIC database, as well as 11 novel mutations (in red) detected in FUSCC cohort.

Supplementary Figure 3

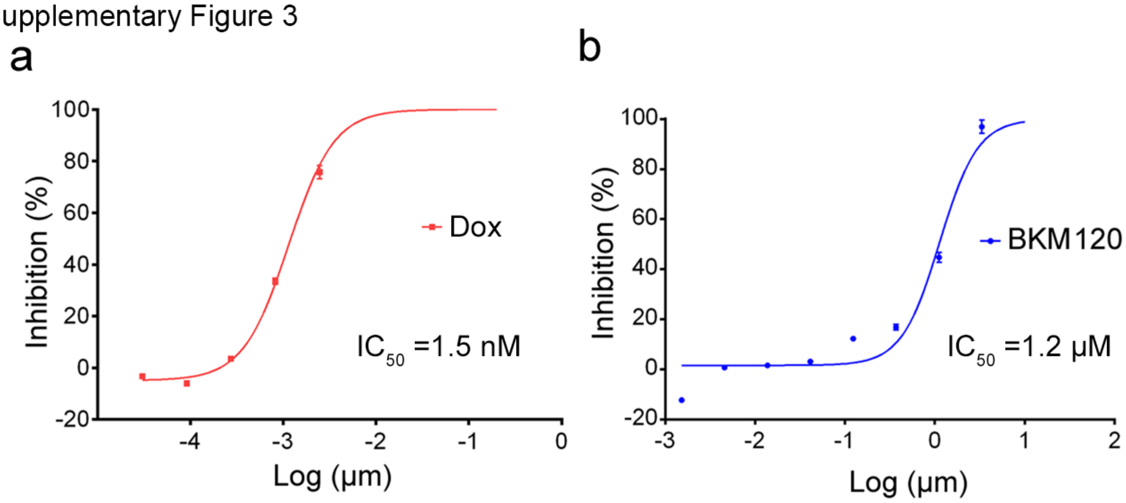

Supplementary Figure 3. The IC_{50} curves for doxorubicin (Dox) **(a)** and BKM120 **(b)** in MCF-10A cell line.

The error bars indicate mean ± s.d. derived from three independent experiments.

Supplementary Figure 4

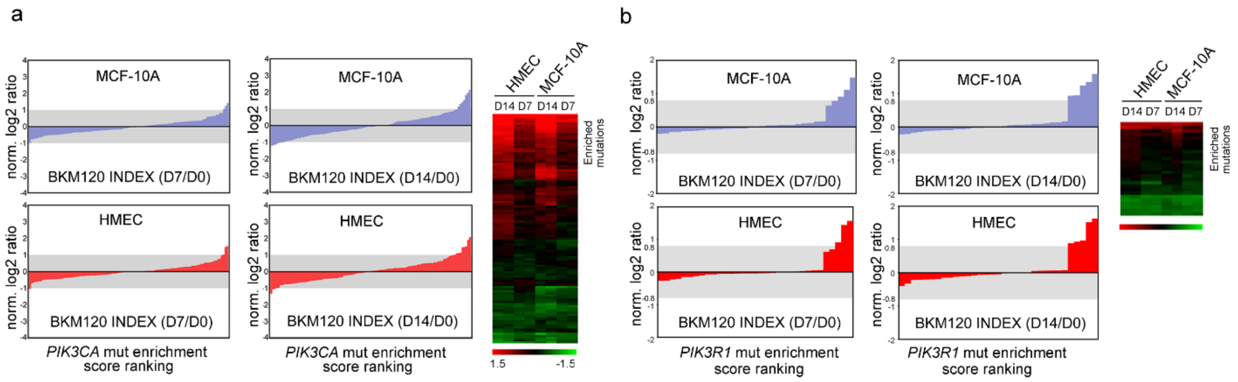

Supplementary Figure 4. ReMB library screening for functional *PIK3CA* and *PIK3R1* mutations based on BKM120 responses in MCF-10A and HEMC cells. **(a)** Waterfall chart showing the enrichment of *PIK3CA* mutations in the indicated assays normalized to Day 0 read counts in MCF-10A and HMEC cells. For each cell line, the *PIK3CA* mutations were ranked on the basis of the mean normalized $\log_2$ (Day 14/Day 0) ratios and $\log_2$ (Day 7/Day 0) ratios of the read counts. The shaded rectangle indicates a $\log_2$ ratio range between -1 to 1. Clustering of the two cell lines reveals the mutations that were consistent between the proliferation and drug response screens. **(b)** Waterfall chart and Clustering show the enrichment of *PIK3R1* mutations in response to BKM120 treatment.

Supplementary Figure 5

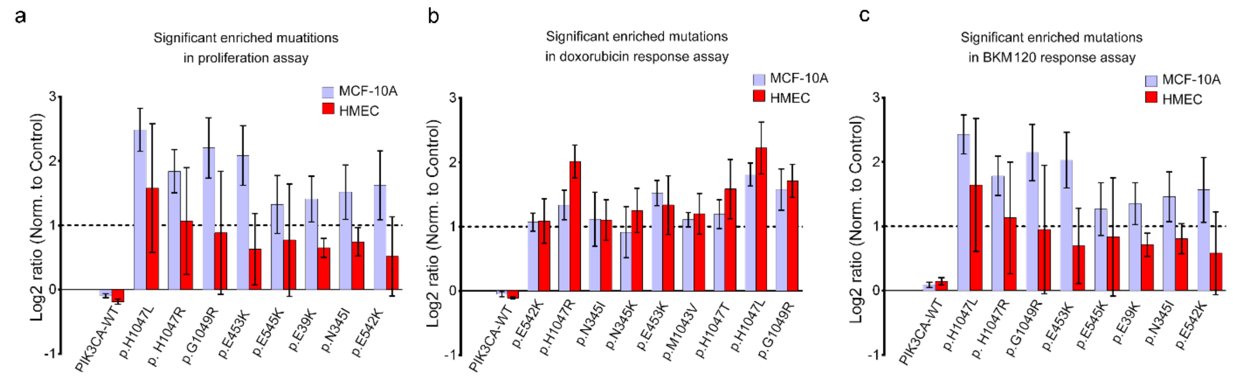

Supplementary Figure 5. The enriched *PIK3CA* mutations were identified in the functional screens. The average enrichment score (Avg ES) indicates the average of two biological replicates; Avg ES was subsequently normalized to the level in the negative controls. An impactful mutation was defined as a mutation with a mean Avg ES greater than 1 in both the MCF-10A and HMEC cell lines. **(a)** Proliferation assay, **(b)** Doxorubicin response, **(c)** BKM120 response.

Supplementary Figure 6

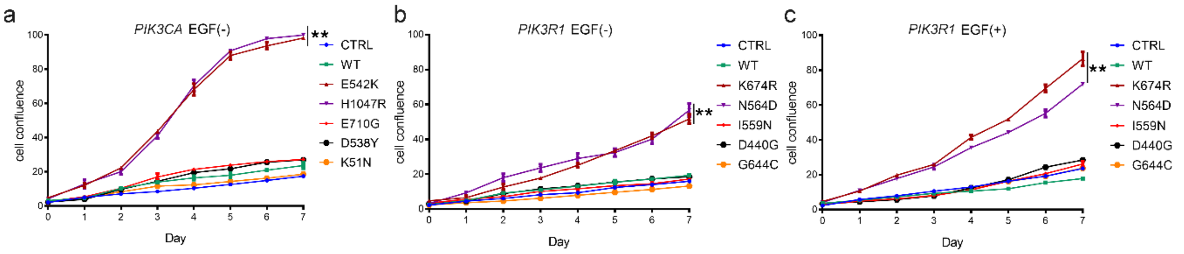

Supplementary Figure 6. Growth curves of MCF-10A cells expressing *PIK3CA* and *PIK3R1* wild type, impactful mutations and non-impactful mutations determined using incucyte cell imaging system. **(a)** Growth curves of cells expressing *PIK3CA* wild type, impactful mutations and non-impactful mutations in the absence of EGF. **(b)** Growth curves of cells expressing *PIK3R1* wild type, impactful mutations and non-impactful mutations in the absence of EGF. **(c)** Growth curves of cells expressing *PIK3R1* wild type, impactful mutations and non-impactful mutations with EGF. Five wells were measured per condition. The error bars shown are mean ± SD of 5 technical replicates from a representative of three independent experiments. (**$P < 0.01$, for Student's *t*-test)

Supplementary Figure 7

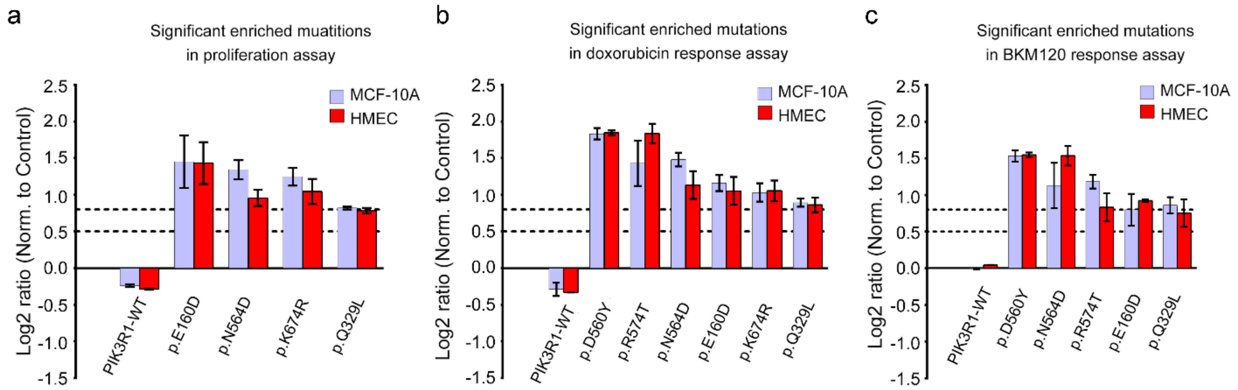

Supplementary Figure 7. The enriched *PIK3R1* mutations were identified in the functional screening assays.

(a) Proliferation assay, **(b)** Doxorubicin response, **(c)** BKM120 response.

Supplementary Figure 8

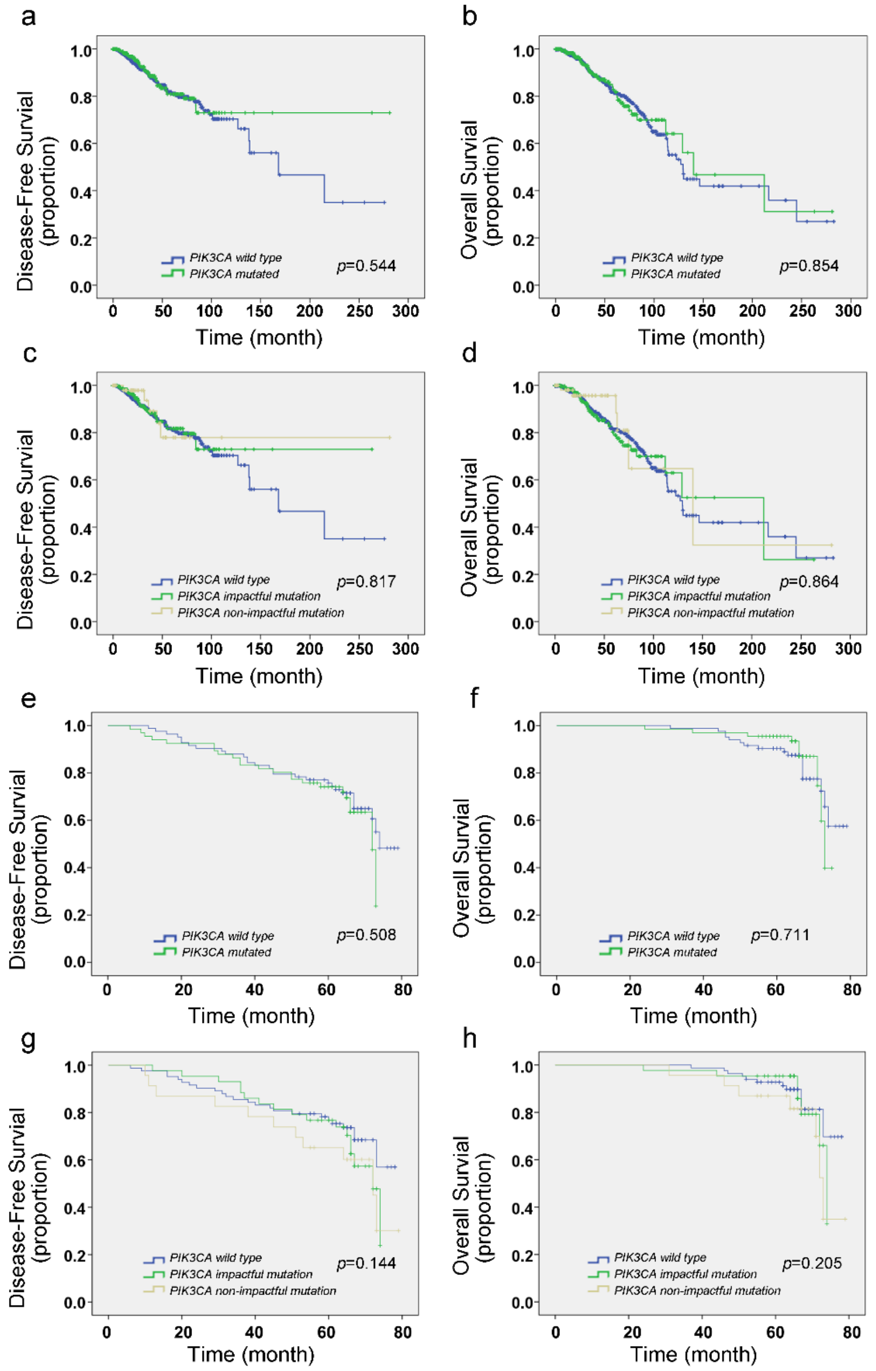

Supplementary Figure 8. Kaplan-Meier estimates of disease-free survival (DFS) and overall survival (OS) by *PIK3CA* alteration in TCGA dataset and FUSCC cohort. **(a,b)** DFS and OS by *PIK3CA* mutation status in TCGA dataset. **(c,d)** DFS and OS by *PIK3CA* impactful mutation status in TCGA dataset. **(e,f)** DFS and OS by *PIK3CA* mutation status in FUSCC cohort. **(g,h)** DFS and OS by *PIK3CA* impactful mutation status in TCGA dataset.

Supplementary Figure 9

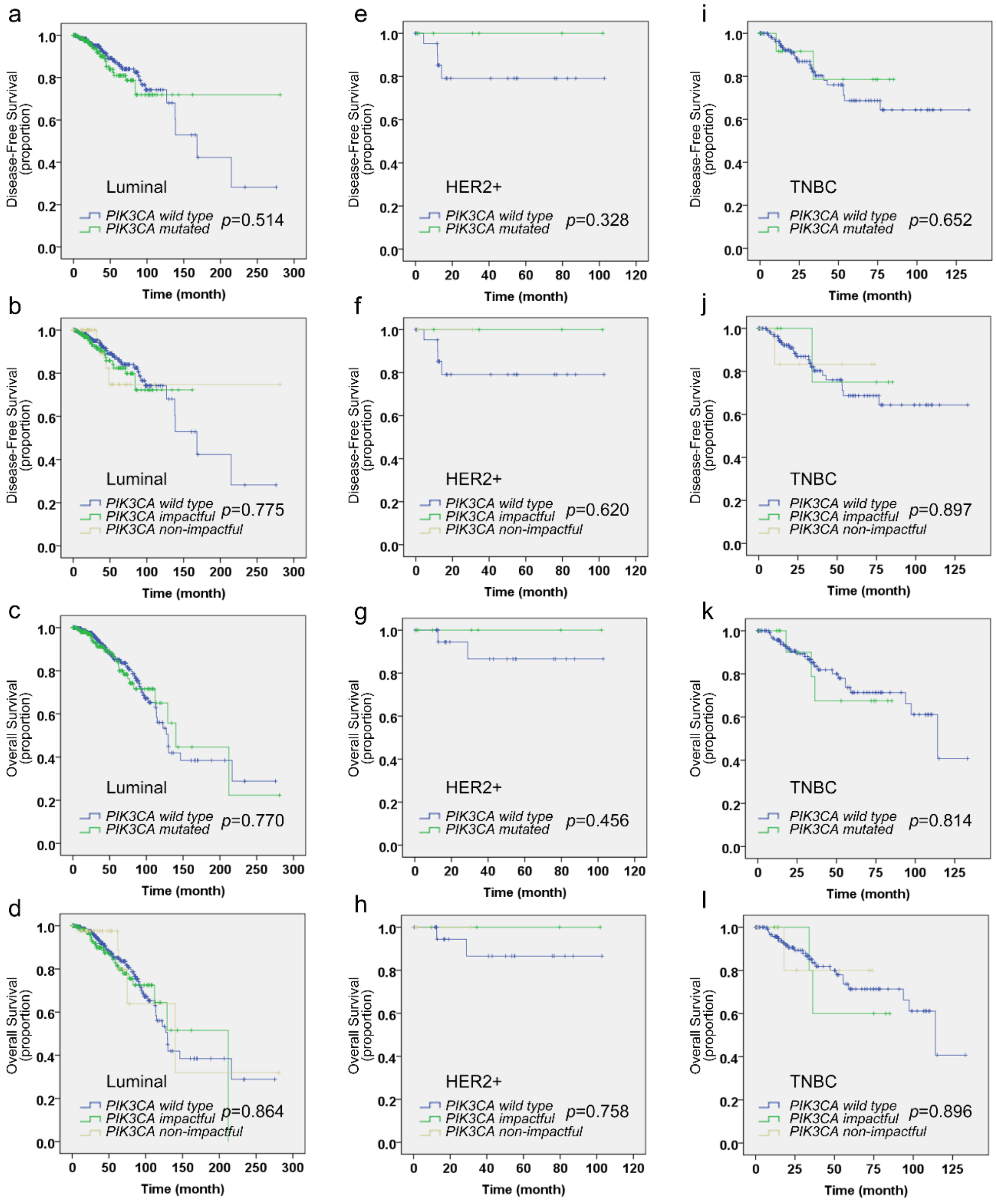

Supplementary Figure 9. Kaplan-Meier estimates of disease-free survival (DFS) and overall survival (OS) by *PIK3CA* alteration in TCGA dataset in different molecular subtypes. **(a-d)** DFS and OS by *PIK3CA* mutation status in luminal subtype. **(e-h)** DFS and OS by *PIK3CA* mutation status in HER2-enriched subtype. **(i-l)** DFS and OS by *PIK3CA* mutation status in TNBC subtype.

Supplementary Figure 10

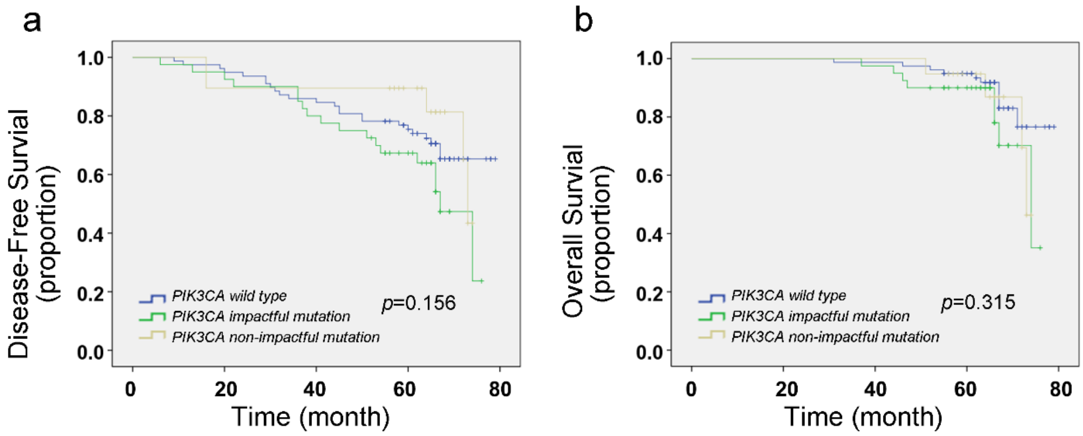

Supplementary Figure 10. Kaplan-Meier estimates of **(a)** disease-free survival (DFS) and **(b)** overall survival (OS) by *PIK3CA* impactful mutation status for patients with doxorubicin treatment in the FUSCC cohort.

Supplementary Figure 11

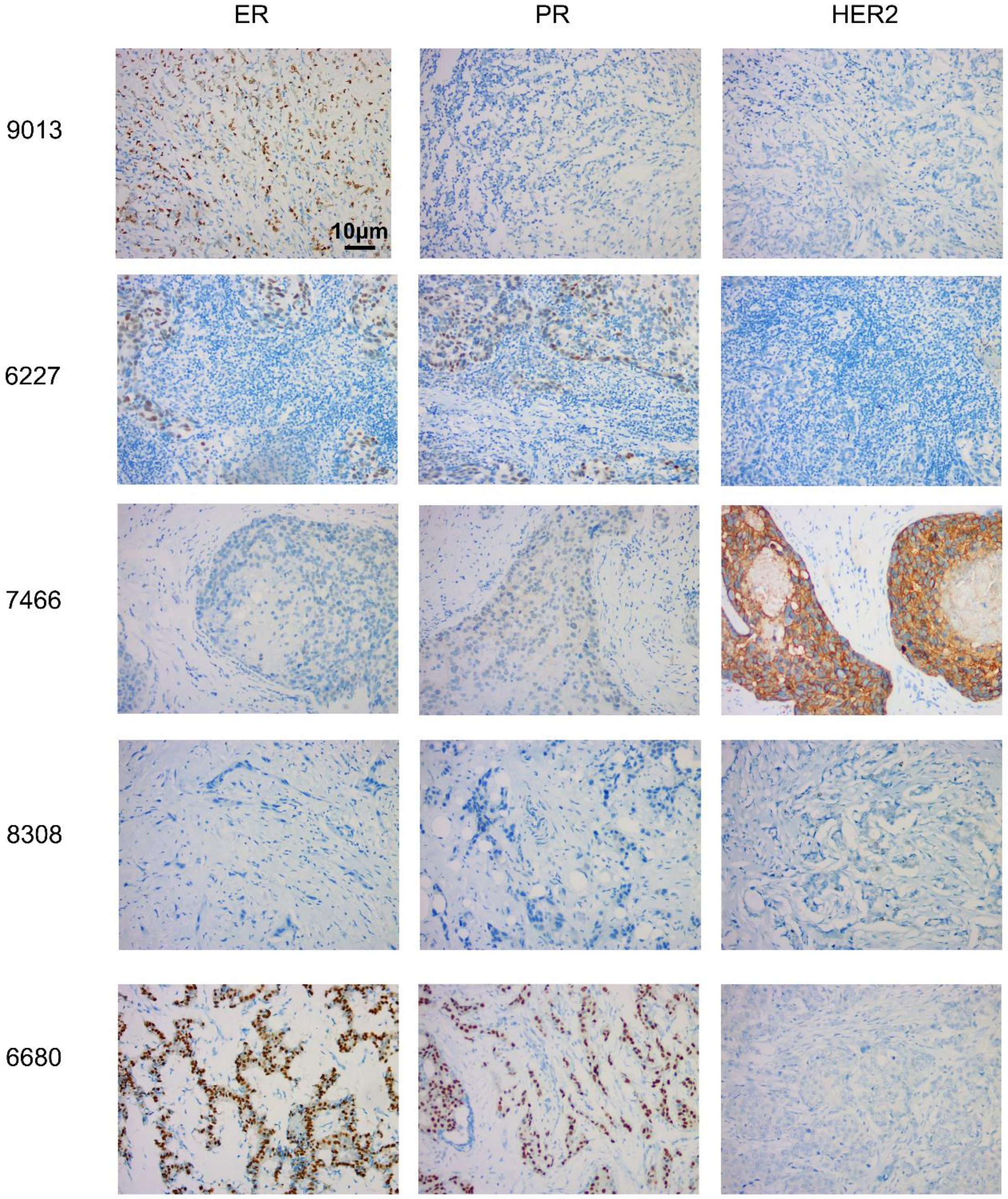

Supplementary Figure 11. Representatives of IHC staining for ER, PR and HER2. Scale bar, 10 μm.

Supplementary Figure 12

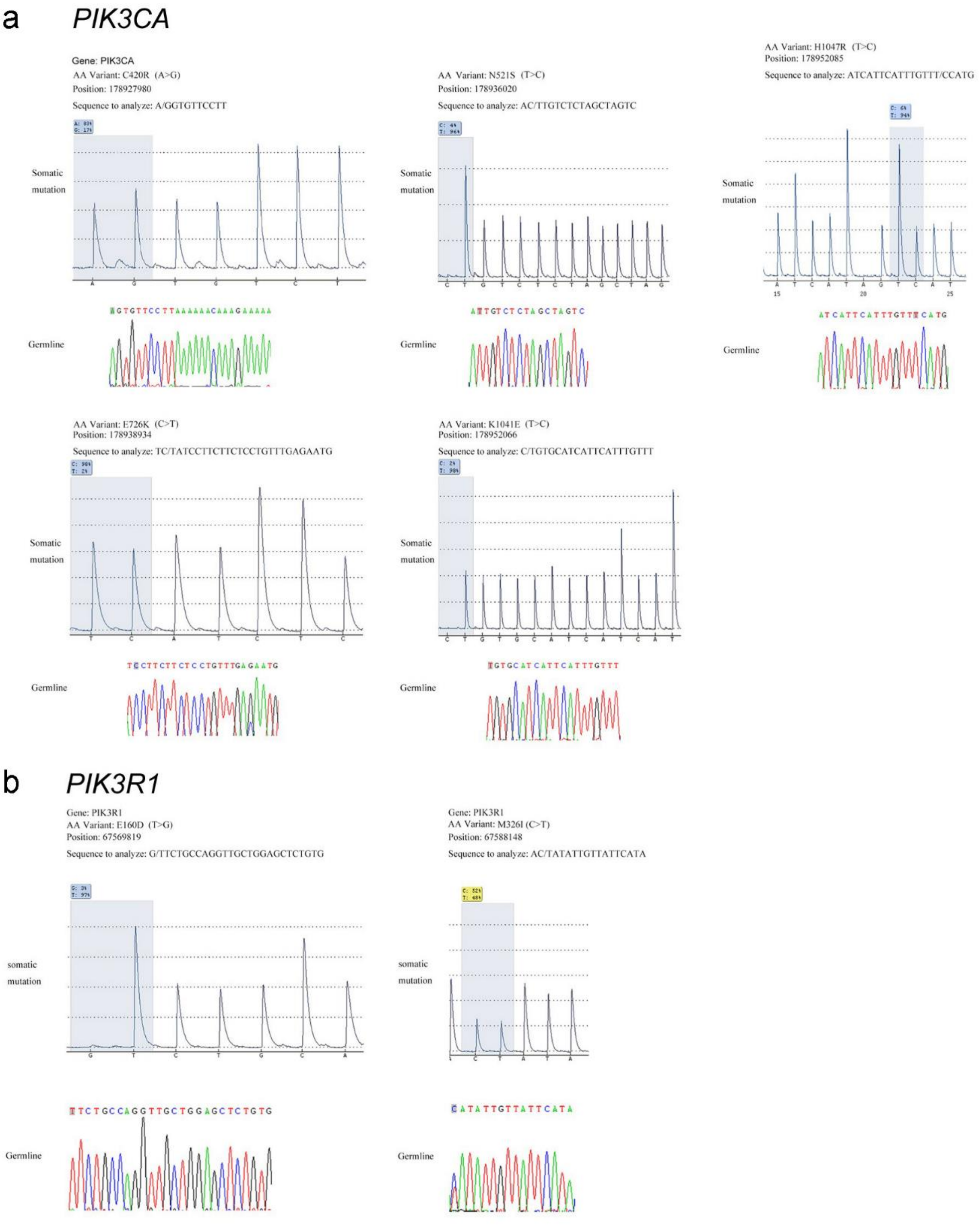

Supplementary Figure 12. Validation of *PIK3CA* and *PIK3R1* somatic mutations. **(a)** Pyrosequencing of malignant tissues and Sanger sequencing of blood DNA for *PIK3CA* mutations. **(b)** Pyrosequencing of malignant tissues and Sanger sequencing of blood DNA for *PIK3R1* mutations.

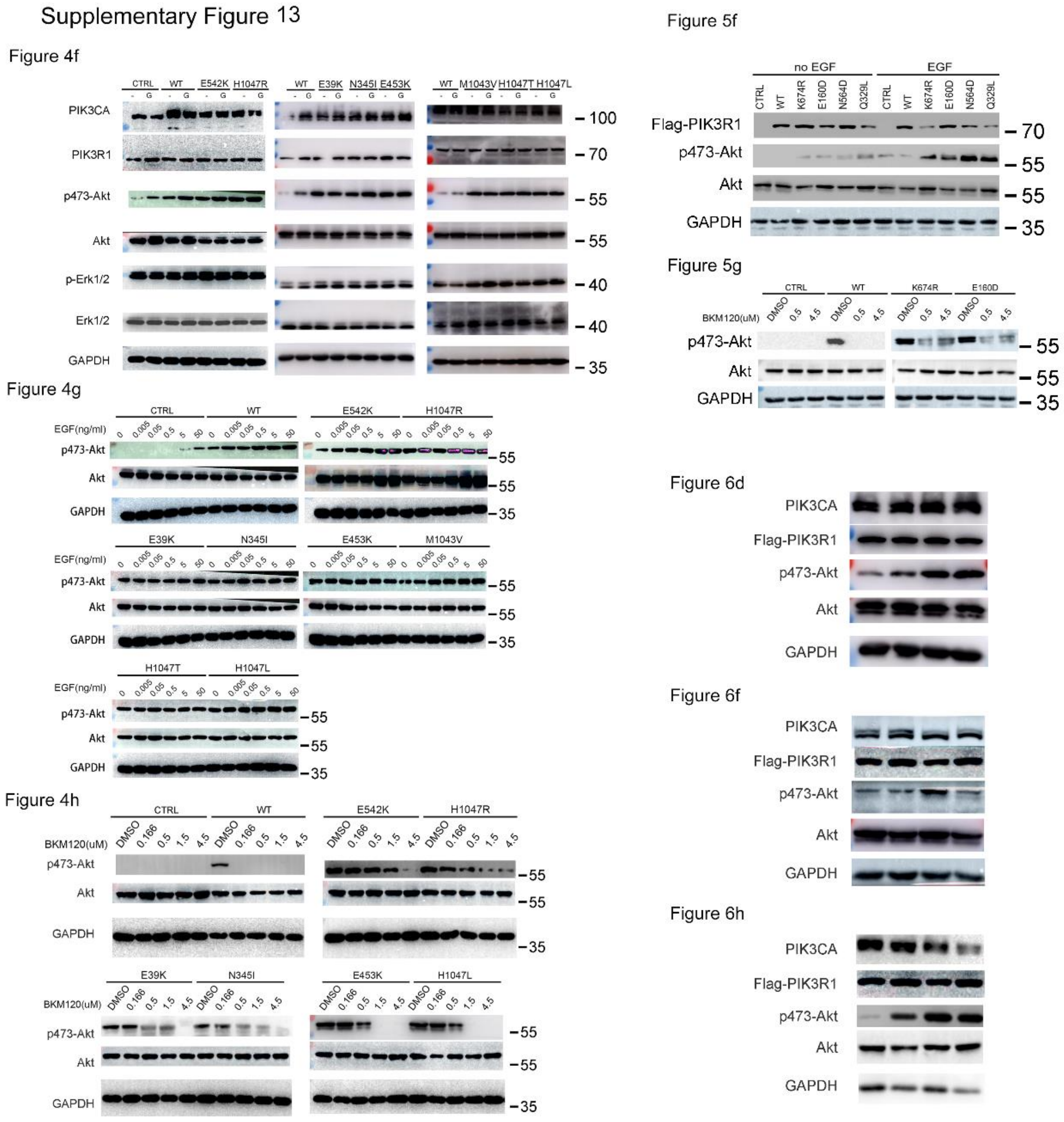

Supplementary Figure 13. Uncropped immunoblot images shown in the manuscript. Figure number and antibody used for the blots are indicated.

Supplementary Table 1 Clinicopathological variables and the mutation status of genes in PI3K/AKT signalling pathway in 149 patients with breast cancer

Variables	Number of patients(%)	PIK3CA mutation		*P* value	PIK3R1 mutation		*P* value	AKT1 mutation		*P* value	AKT2 mutation		*P* value	AKT3 mutation		*P* value	PDK1 mutation		*P* value	PTEN mutation		value
		Neg n (%)	Pos n (%)		Neg n (%)	Pos n (%)		Neg n (%)	Pos n (%)		Neg n (%)	Pos n (%)		Neg n (%)	Pos n (%)		Neg n (%)	Pos n (%)		Neg n (%)	Pos n (%)	
Total	149	84(56.4)	65(43.6)		124(83.2)	25(16.8)		134(89.9)	15(10.1)		134(89.9)	15(10.1)		127(85.2)	22(14.8)		142(95.3)	7(4.7)		131(87.9)	18(12.1)	
Age (median 51, range 25-72)				0.463			0.395			0.845			0.066			0.294			0.139			0.305
≤50 years	83(55.7)	49(32.9)	34(22.8)		71(47.7)	12(8.1)		75(50.3)	8(5.4)		78(52.3)	5(3.4)		73(49)	10(6.7)		81(54.4)	2(1.3)		75(50.3)	8(5.4)	
>50years	66(44.3)	35(23.5)	31(20.8)		53(35.6)	13(8.7)		59(39.6)	7(4.7)		56(37.6)	10(6.7)		54(36.2)	12(8.1)		61(40.9)	5(3.4)		56(37.6)	10(6.7)	
status				0.884			0.785			0.357			0.184			0.075			0.768			0.903
Premenopause	93(62.4)	52(34.9)	41(27.5)		78(52.3)	15(10.1)		86(57.7)	7(4.7)		86(57.7)	7(4.7)		83(55.7)	10(6.7)		89(59.7)	4(2.7)		82(55.0)	11(7.4)	
Postmenopause	56(37.6)	32(21.5)	24(16.1)		46(30.9)	10(6.7)		48(32.2)	8(5.4)		48(32.2)	8(5.4)		44(29.5)	12(8.1)		53(35.6)	3(2.0)		49(32.9)	7(4.7)	
Tumor size				0.196			0.831			0.385			0.385			0.121			0.325			0.492
≤2cm	79(53.0)	49(32.9)	30(20.1)		65(43.6)	14(9.4)		73(49.0)	6(4.0)		73(49)	6(4.0)		71(47.7)	8(5.4)		77(51.7)	2(1.3)		71(47.7)	8(5.4)	
>2, 5≤cm	66(44.3)	34(22.8)	32(21.5)		56(37.6)	10(6.7)		57(38.3)	9(6.0)		57(38.3)	9(6.0)		52(34.9)	14(9.4)		61(40.9)	5(3.4)		56(37.6)	10(6.7)	
>5cm	4(2.7)	1(0.7)	3(2.0)		3(2)	1(0.7)		4(2.7)	0(0)		4(2.7)	0(0)		4(2.7)	0(0)		4(2.7)	0(0)		4(2.7)	0(0)	
LN status				0.845			**0.008**			0.221			0.892			**0.032**			0.632			0.175
Negative	72(48.3)	40(26.8)	32(21.5)		66(44.3)	6(4.0)		67(45.0)	5(3.4)		65(43.6)	7(4.7)		66(44.3)	6(4.0)		68(45.6)	4(2.7)		66(44.3)	6(4.0)	
Positive	77(51.7)	44(29.5)	33(22.1)		58(38.9)	19(12.8)		67(45.0)	10(6.7)		69(46.3)	8(5.4)		61(40.9)	16(10.7)		74(49.7)	3(2.0)		65(43.6)	12(8.1)	
Grade				0.308			0.334			**0.048**			0.529			0.398			0.312			0.503
1	9(6.0)	3(2.0)	6(4.0)		8(5.4)	1(0.7)		6(4.0)	3(2.0)		9(6.0)	0(0)		9(6.0)	0(0)		8(5.4)	1(0.7)		9(6.0)	0(0)	
2	82(55.1)	49(32.9)	33(22.2)		71(47.7)	11(7.4)		76(51.0)	6(4.0)		74(49.7)	8(5.4)		70(47)	12(8.1)		77(51.7)	5(3.4)		71(47.7)	11(7.4)	
3	58(38.9)	32(21.5)	26(17.4)		45(30.2)	13(8.7)		52(34.9)	6(4.0)		51(34.2)	7(4.7)		48(32.2)	10(6.7)		57(38.3)	1(0.7)		51(34.2)	7(4.7)	
ER status				0.060			**0.035**			0.147			0.798			**0.023**			0.955			0.706
Negative	44(29.5)	30(20.1)	14(9.4)		41(27.5)	3(2.0)		42(28.2)	2(1.3)		40(26.8)	4(2.7)		42(28.2)	2(1.3)		42(28.2)	2(1.3)		38(25.5)	6(4)	
Positive	105(70.5)	54(36.3)	51(34.2)		83(55.7)	22(14.8)		92(61.7)	13(8.8)		94(63.1)	11(7.4)		85(57)	20(13.4)		100(67.1)	5(3.4)		93(62.4)	12(8.1)	
PR status				0.464			0.185			0.448			0.344			**0.065**			0.692			0.173
Negative	53(35.6)	32(21.5)	21(14.1)		47(31.5)	6(4.0)		49(32.9)	4(2.7)		46(30.9)	7(4.7)		49(32.9)	4(2.7)		51(34.2)	2(1.3)		44(29.5)	9(6.0)	
Positive	96(64.4)	52(34.9)	44(29.5)		77(51.7)	19(12.8)		85(57)	11(7.4)		88(59.1)	8(5.4)		78(52.3)	18(12.1)		91(61.1)	5(3.4)		87(58.4)	9(6.0)	
status				0.730			0.421			0.762			0.762			0.674			0.738			0.480
Negative	94(63.1)	54(36.2)	40(26.8)		80(53.7)	14(9.4)		84(56.4)	10(6.7)		84(56.4)	10(6.7)		81(54.4)	13(8.7)		90(60.4)	4(2.7)		84(56.4)	10(6.7)	
Positive	55(36.9)	30(20.1)	25(16.8)		44(29.5)	11(7.4)		50(33.6)	5(3.4)		50(33.6)	5(3.4)		46(30.9)	9(6.0)		52(34.9)	3(2.0)		47(31.5)	8(5.4)	
LVI				0.477			**0.037**			**0.047**			0.849			0.051			0.739			0.553
Negative	76(51.0)	45(30.2)	31(20.8)		68(45.6)	8(5.4)		72(48.3)	4(2.7)		68(45.6)	8(5.4)		69(46.3)	7(4.7)		72(48.3)	4(2.7)		68(45.6)	8(5.4)	
Positive	73(49.0)	39(26.2)	34(22.8)		56(37.6)	17(11.4)		62(41.6)	11(7.4)		66(44.3)	7(4.7)		58(38.9)	15(10.1)		70(47.0)	3(2.0)		63(42.3)	10(6.7)	

Abbreviations: ER, estrogen receptor; HER-2, human epidermal growth factor receptor 2; PR, progesterone receptor; SD, standard deviation; Neg, negative; Pos, positive; LN, lymph node; LVI,lymphatic vessel invasion.

Note: Based on Pearson χ2 test except for surgery type and radiation therapy, for which P is based on Fisher's exact test; Bold values denote *P* value < 0.05.

Supplementary Table 2 Summary of amplicon sequencing readout for genes in PI3K/AKT pathway using Ion Torrent platform

Patient ID	Gene	Chrom	Position	Ref	Variant	Coverage	Frequency (%)	AA Change
4487	*AKT1*	chr14	105242077	T	C	154	4.5	E116G
4516	*AKT1*	chr14	105236736	T	C	232	3.4	D462G
6050	*AKT1*	chr14	105246551	C	T	1074	17.5	E17K
6227	*AKT1*	chr14	105246551	C	T	1635	18.3	E17K
6670	*AKT1*	chr14	105246551	C	T	1238	17.7	E17K
6680	*AKT1*	chr14	105239326	T	C	18	11.1	H354R
6740	*AKT1*	chr14	105240307	T	A	151	3.3	Y215F
6980	*AKT1*	chr14	105241501	G	C	420	4	T160S
9708	*AKT1*	chr14	105246551	C	T	1346	14	E17K
2226	*AKT1*	chr14	105236736	T	C	197	11.2	D462G
2226	*AKT1*	chr14	105242077	T	C	116	6	E116G
6392	*AKT1*	chr14	105239323	T	C	27	7.4	E355G
6768	*AKT1*	chr14	105236736	T	C	444	3.2	D462G
8450	*AKT1*	chr14	105246551	C	T	848	4.1	E17K
0585	*AKT1*	chr14	105236736	T	C	440	4.8	D462G
2124	*AKT1*	chr14	105236748	A	G	791	39.4	M458T
9714	*AKT1*	chr14	105246551	C	T	1991	42.9	E17K
9714	*AKT1*	chr14	105246551	C	T	1998	42.9	E17K
9714	*AKT1*	chr14	105246551	C	T	1998	41.3	E17K
1102	*AKT1*	chr14	105246551	C	T	986	71.2	E17K
2347	*AKT1*	chr14	105246445	A	T	1974	11	L52H
2012	*AKT2*	chr19	40743884	C	T	1999	6.6	D275N
4487	*AKT2*	chr19	40740981	T	A	101	8.9	Q446L
4516	*AKT2*	chr19	40740981	T	A	112	17.9	Q446L
4952	*AKT2*	chr19	40740981	T	A	228	9.2	Q446L
6740	*AKT2*	chr19	40740981	T	A	118	10.2	Q446L
6740	*AKT2*	chr19	40740994	C	T	128	3.9	E442K
2226	*AKT2*	chr19	40740981	T	A	58	25.9	Q446L
4377	*AKT2*	chr19	40740963	G	T	113	7.1	P452H
4377	*AKT2*	chr19	40740981	T	A	147	10.9	Q446L
4377	*AKT2*	chr19	40740994	C	T	154	5.8	E442K
6705	*AKT2*	chr19	40740981	T	A	252	6.7	Q446L
6768	*AKT2*	chr19	40740981	T	A	118	9.3	Q446L
6768	*AKT2*	chr19	40740994	C	T	126	6.3	E442K
7717	*AKT2*	chr19	40740981	T	A	145	10.3	Q446L
9310	*AKT2*	chr19	40741225	A	G	51	5.9	V403A
0585	*AKT2*	chr19	40740981	T	A	122	18	Q446L
0585	*AKT2*	chr19	40740994	C	T	131	6.9	E442K
1590	*AKT2*	chr19	40740981	T	A	157	12.7	Q446L
8720	*AKT2*	chr19	40740981	T	A	219	3.2	Q446L
9444	*AKT2*	chr19	40740981	T	A	85	11.8	Q446L
4487	*AKT3*	chr1	243727086	T	C	817	3.9	E295G
4487	*AKT3*	chr1	243727096	G	T	759	4.5	L292I
4516	*AKT3*	chr1	243727086	T	C	746	3.2	E295G
6740	*AKT3*	chr1	243727086	T	C	814	3.2	E295G
6740	*AKT3*	chr1	243727096	G	T	762	3.3	L292I
2226	*AKT3*	chr1	243727086	T	C	598	8.7	E295G
2226	*AKT3*	chr1	243727096	G	T	493	11	L292I
6705	*AKT3*	chr1	243777013	T	C	27	7.4	D219G
6768	*AKT3*	chr1	243727086	T	C	1130	3.5	E295G
7717	*AKT3*	chr1	243727086	T	C	1209	3.6	E295G
0585	*AKT3*	chr1	243727086	T	C	787	3.7	E295G
5858	*AKT3*	chr1	243828153	G	T	607	3.6	P69T
6319	*AKT3*	chr1	243736334	G	A	1374	3.6	S238L
6319	*AKT3*	chr1	243828153	G	T	841	4	P69T
6319	*AKT3*	chr1	243828153	G	T	1472	3.7	P69T
6344	*AKT3*	chr1	243828153	G	T	742	4.6	P69T
6344	*AKT3*	chr1	243828153	G	T	799	6	P69T
6344	*AKT3*	chr1	243828153	G	T	1997	4.2	P69T
6864	*AKT3*	chr1	243736334	G	A	2000	5.5	S238L
7509	*AKT3*	chr1	243736334	G	A	2000	5.5	S238L
8153	*AKT3*	chr1	243736334	G	A	1999	5.6	S238L
8153	*AKT3*	chr1	243736334	G	A	1999	3.9	S238L
8176	*AKT3*	chr1	243736334	G	A	1443	9.3	S238L
8177	*AKT3*	chr1	244006460	T	A	1996	4.7	T5S
8408	*AKT3*	chr1	243736334	G	A	1999	5.2	S238L
8408	*AKT3*	chr1	244006460	T	A	1917	3.9	T5S
1151	*PDK1*	chr2	173435485	C	G	1326	50.1	A313G
8500	*PDK1*	chr2	173451103	G	A	846	20.1	R368H
2226	*PDK1*	chr2	173451012	G	A	113	10.6	D338N
6392	*PDK1*	chr2	173460575	A	G	2000	48	I417V
9714	*PDK1*	chr2	173429330	T	A	1999	3.8	D170E
0984	*PDK1*	chr2	173451022	G	A	1082	4.1	G321D
2793	*PDK1*	chr2	173435472	A	G	1998	3	M289V
2407	*PIK3CA*	chr3	178919171	A	G	645	3.1	Q219R
2407	*PIK3CA*	chr3	178952085	A	G	1497	18.8	H1047R
1151	*PIK3CA*	chr3	178919171	A	G	432	11.8	Q219R
9196	*PIK3CA*	chr3	178919171	A	G	426	5.6	Q219R
0189	*PIK3CA*	chr3	178952085	A	G	1373	31.8	H1047R
2012	*PIK3CA*	chr3	178919171	A	G	561	5.2	Q219R
4155	*PIK3CA*	chr3	178952085	A	G	1509	12.6	H1047R
4362	*PIK3CA*	chr3	178919171	A	G	507	3.6	Q219R
4487	*PIK3CA*	chr3	178919171	A	G	198	41.9	Q219R
4487	*PIK3CA*	chr3	178941960	A	T	990	4.5	Q760L
4487	*PIK3CA*	chr3	178952066	A	G	537	8.8	K1041E
4516	*PIK3CA*	chr3	178919171	A	G	122	19.7	Q219R
4516	*PIK3CA*	chr3	178941960	A	T	675	5.6	Q760L
4516	*PIK3CA*	chr3	178952066	A	G	440	6.1	K1041E
4952	*PIK3CA*	chr3	178919171	A	G	279	21.9	Q219R

4983	*PIK3CA*	chr3	178952085	A	G	1730	4.6	H1047R
5026	*PIK3CA*	chr3	178919171	A	G	446	11.2	Q219R
5026	*PIK3CA*	chr3	178952085	A	G	1242	19.7	H1047R
5831	*PIK3CA*	chr3	178919171	A	G	492	4.9	Q219R
6227	*PIK3CA*	chr3	178919171	A	G	494	3.8	Q219R
6670	*PIK3CA*	chr3	178919171	A	G	317	4.1	Q219R
6680	*PIK3CA*	chr3	178935998	A	G	19	10.5	S514G
6740	*PIK3CA*	chr3	178916766	A	T	253	5.5	K51N
6740	*PIK3CA*	chr3	178919171	A	G	276	42	Q219R
6740	*PIK3CA*	chr3	178941960	A	T	1152	3.6	Q760L
6740	*PIK3CA*	chr3	178952066	A	G	698	8.5	K1041E
6740	*PIK3CA*	chr3	178952085	A	G	716	45.9	H1047R
7022	*PIK3CA*	chr3	178916946	G	T	1996	70.7	K111N
7022	*PIK3CA*	chr3	178952085	A	G	1870	70.7	H1047R
7061	*PIK3CA*	chr3	178952085	A	G	1318	22.5	H1047R
7466	*PIK3CA*	chr3	178919171	A	G	567	3	Q219R
9013	*PIK3CA*	chr3	178919171	A	G	771	5.1	Q219R
9114	*PIK3CA*	chr3	178916728	G	A	779	40.2	E39K
9557	*PIK3CA*	chr3	178919171	A	G	247	21.9	Q219R
9557	*PIK3CA*	chr3	178952085	A	G	936	3.4	H1047R
9590	*PIK3CA*	chr3	178952085	A	G	1336	48.4	H1047R
9828	*PIK3CA*	chr3	178919171	A	G	534	7.5	Q219R
9841	*PIK3CA*	chr3	178916701	A	G	395	5.1	M30V
9841	*PIK3CA*	chr3	178919171	A	G	296	24.3	Q219R
0766	*PIK3CA*	chr3	178919171	A	G	599	5	Q219R
0916	*PIK3CA*	chr3	178936020	A	G	26	11.5	N521S
0916	*PIK3CA*	chr3	178938934	G	A	1601	6.5	E726K
2226	*PIK3CA*	chr3	178916766	A	T	112	5.4	K51N
2226	*PIK3CA*	chr3	178916840	A	G	111	4.5	E76G
2226	*PIK3CA*	chr3	178919171	A	G	194	53.6	Q219R
2226	*PIK3CA*	chr3	178928306	T	C	471	3.8	W498R
2226	*PIK3CA*	chr3	178938887	A	G	549	3.1	E710G
2226	*PIK3CA*	chr3	178941960	A	T	640	15.2	Q760L
2226	*PIK3CA*	chr3	178952066	A	G	533	13.5	K1041E
4377	*PIK3CA*	chr3	178916766	A	T	150	7.3	K51N
4377	*PIK3CA*	chr3	178919147	T	G	88	3.4	I211S
4377	*PIK3CA*	chr3	178919171	A	G	164	35.4	Q219R
4377	*PIK3CA*	chr3	178941960	A	T	513	3.9	Q760L
4377	*PIK3CA*	chr3	178952066	A	G	401	5.7	K1041E
4707	*PIK3CA*	chr3	178936091	G	A	2000	16.7	E545K
4998	*PIK3CA*	chr3	178936082	G	A	1077	23.1	E542K
5342	*PIK3CA*	chr3	178952085	A	T	1480	23.9	H1047R
6023	*PIK3CA*	chr3	178936091	G	A	1998	31.6	E545K
6113	*PIK3CA*	chr3	178919171	A	G	959	8.4	Q219R
6179	*PIK3CA*	chr3	178952085	A	G	2000	72.7	H1047R
6392	*PIK3CA*	chr3	178952085	A	G	1523	38.5	H1047R
6705	*PIK3CA*	chr3	178919171	A	G	201	14.4	Q219R
6768	*PIK3CA*	chr3	178916701	A	G	325	3.1	M30V
6768	*PIK3CA*	chr3	178916766	A	T	348	3.2	K51N
6768	*PIK3CA*	chr3	178919171	A	G	310	32.6	Q219R
6768	*PIK3CA*	chr3	178941960	A	T	1546	3.3	Q760L
6768	*PIK3CA*	chr3	178952066	A	G	857	5.5	K1041E
6768	*PIK3CA*	chr3	178952085	A	G	876	16.9	H1047R
7030	*PIK3CA*	chr3	178952085	A	G	1354	9.5	H1047R
7717	*PIK3CA*	chr3	178916701	A	G	213	3.8	M30V
7717	*PIK3CA*	chr3	178916766	A	T	237	3.4	K51N
7717	*PIK3CA*	chr3	178919119	A	G	117	3.4	N202D
7717	*PIK3CA*	chr3	178919171	A	G	278	37.4	Q219R
7717	*PIK3CA*	chr3	178941960	A	T	1066	3.2	Q760L
7717	*PIK3CA*	chr3	178952066	A	G	700	2.9	K1041E
8364	*PIK3CA*	chr3	178919171	A	G	491	3.9	Q219R
9410	*PIK3CA*	chr3	178919171	A	G	572	5.2	Q219R
9410	*PIK3CA*	chr3	178936091	G	A	1999	22.9	E545K
0585	*PIK3CA*	chr3	178916766	A	T	235	3.4	K51N
0585	*PIK3CA*	chr3	178919171	A	G	195	21.5	Q219R
0585	*PIK3CA*	chr3	178941960	A	T	899	4.9	Q760L
0585	*PIK3CA*	chr3	178952066	A	G	580	6.7	K1041E
0630	*PIK3CA*	chr3	178919171	A	G	637	8	Q219R
1217	*PIK3CA*	chr3	178919171	A	G	333	4.2	Q219R
1365	*PIK3CA*	chr3	178919171	A	G	524	3.4	Q219R
1365	*PIK3CA*	chr3	178952085	A	T	1254	33.2	H1047R
1590	*PIK3CA*	chr3	178916701	A	G	263	3	M30V
1590	*PIK3CA*	chr3	178919171	A	G	288	27.1	Q219R
2124	*PIK3CA*	chr3	178919171	A	G	465	4.7	Q219R
3108	*PIK3CA*	chr3	178952085	A	G	1448	30	H1047R
5321	*PIK3CA*	chr3	178952085	A	G	1294	26.8	H1047R
5324	*PIK3CA*	chr3	178936091	G	A	2000	11.1	E545K
5409	*PIK3CA*	chr3	178927980	T	C	1020	17.5	C420R
5598	*PIK3CA*	chr3	178936004	A	G	17	11.8	R516G
5925	*PIK3CA*	chr3	178952085	A	G	1417	12.3	H1047R
6433	*PIK3CA*	chr3	178936094	C	A	1999	3.7	Q546K
8346	*PIK3CA*	chr3	178952085	A	G	1370	6.1	H1047R
8720	*PIK3CA*	chr3	178916701	A	G	483	3.1	M30V
8720	*PIK3CA*	chr3	178919171	A	G	399	15.8	Q219R
9080	*PIK3CA*	chr3	178921553	T	A	1797	22.6	N345K
9190	*PIK3CA*	chr3	178921553	T	A	1997	13.5	N345K
9238	*PIK3CA*	chr3	178936091	G	A	2000	11.7	E545K
9444	*PIK3CA*	chr3	178916701	A	G	142	4.2	M30V
9444	*PIK3CA*	chr3	178916766	A	T	160	5.6	K51N
9444	*PIK3CA*	chr3	178919171	A	G	165	32.7	Q219R
9444	*PIK3CA*	chr3	178941960	A	T	567	4.2	Q760L

9444	*PIK3CA*	chr3	178952066	A	G	407	7.4	K1041E
9444	*PIK3CA*	chr3	178952085	A	G	413	39	H1047R
9589	*PIK3CA*	chr3	178928117	T	G	1984	6.1	N465K
9714	*PIK3CA*	chr3	178936082	G	A	1994	41.5	E542K
9714	*PIK3CA*	chr3	178936091	G	A	2000	19	E545K
9714	*PIK3CA*	chr3	178936091	G	A	2000	24.4	E545K
0984	*PIK3CA*	chr3	178936091	G	A	2000	22	E545K
0984	*PIK3CA*	chr3	178952090	G	C	1997	12	G1049R
1413	*PIK3CA*	chr3	178952085	A	G	2000	8.8	H1047R
1432	*PIK3CA*	chr3	178952085	A	G	1997	9.7	H1047R
2331	*PIK3CA*	chr3	178952085	A	G	1990	41.1	H1047R
2347	*PIK3CA*	chr3	178916946	G	C	2000	19.5	K111N
2347	*PIK3CA*	chr3	178928288	A	G	965	5.5	I492V
2347	*PIK3CA*	chr3	178952085	A	T	1963	31.3	H1047L
2347	*PIK3CA*	chr3	178952085	A	T	1979	17	H1047L
2347	*PIK3CA*	chr3	178952085	A	T	1994	59.1	H1047L
2347	*PIK3CA*	chr3	178952085	A	G	1998	13.7	H1047R
2347	*PIK3CA*	chr3	178952085	A	G	1999	8	H1047R
2368	*PIK3CA*	chr3	178952085	A	G	1996	21.5	H1047R
2368	*PIK3CA*	chr3	178952085	A	G	2000	24.5	H1047R
2440	*PIK3CA*	chr3	178952085	A	G	1995	24.4	H1047R
2440	*PIK3CA*	chr3	178952085	A	G	1998	3.7	H1047R
4487	*PIK3R1*	chr5	67522754	C	T	131	3.8	P84L
4487	*PIK3R1*	chr5	67569819	A	C	397	4.3	E160D
4487	*PIK3R1*	chr5	67591300	A	C	218	3.2	N600H
4516	*PIK3R1*	chr5	67569819	A	C	171	5.3	E160D
4516	*PIK3R1*	chr5	67591287	C	G	93	3.2	N595K
4952	*PIK3R1*	chr5	67591300	A	C	233	4.7	N600H
6740	*PIK3R1*	chr5	67522754	C	T	129	4.7	P84L
9557	*PIK3R1*	chr5	67591300	A	C	341	4.1	N600H
2226	*PIK3R1*	chr5	67522754	C	T	77	6.5	P84L
2226	*PIK3R1*	chr5	67569819	A	C	223	10.3	E160D
2226	*PIK3R1*	chr5	67576359	T	G	141	4.3	V213D
2226	*PIK3R1*	chr5	67591287	C	G	156	4.5	N595K
2226	*PIK3R1*	chr5	67592079	A	T	895	3.2	N632I
4377	*PIK3R1*	chr5	67588156	A	T	367	7.1	Q329L
7717	*PIK3R1*	chr5	67522754	C	T	186	5.4	P84L
9310	*PIK3R1*	chr5	67575459	G	A	29	17.2	D178N
9310	*PIK3R1*	chr5	67591061	C	A	175	33.1	Q552K
9310	*PIK3R1*	chr5	67591083	T	A	180	3.3	I559N
0585	*PIK3R1*	chr5	67569819	A	C	392	5.1	E160D
5598	*PIK3R1*	chr5	67591097	A	G	1234	3.9	N564D
7885	*PIK3R1*	chr5	67593272	A	C	1995	6.6	N673T
7885	*PIK3R1*	chr5	67593272	A	C	1996	4	N673T
9080	*PIK3R1*	chr5	67593272	A	C	1927	7.9	N673T
9080	*PIK3R1*	chr5	67593272	A	C	1997	3.8	N673T
9521	*PIK3R1*	chr5	67593272	A	C	1997	3.5	N673T
9521	*PIK3R1*	chr5	67593272	A	C	1999	3.7	N673T
2793	*PIK3R1*	chr5	67593275	A	G	2000	4.5	K674R
2793	*PIK3R1*	chr5	67593275	A	G	2000	11.2	K674R
4818	*PIK3R1*	chr5	67588148	G	A	1999	50.1	M326I
4830	*PIK3R1*	chr5	67588148	G	A	2000	51.1	M326I
5305	*PIK3R1*	chr5	67588148	G	A	1998	55.1	M326I
5650	*PIK3R1*	chr5	67588148	G	A	2000	48.7	M326I
5706	*PIK3R1*	chr5	67588148	G	A	1998	40	M326I
5722	*PIK3R1*	chr5	67588148	G	A	2000	37.4	M326I
5858	*PIK3R1*	chr5	67588148	G	A	1996	50.1	M326I
8177	*PIK3R1*	chr5	67590448	T	G	1984	7.2	Y504D
8177	*PIK3R1*	chr5	67590448	T	G	1988	5.8	Y504D
8287	*PIK3R1*	chr5	67590448	T	G	1988	5.9	Y504D
8287	*PIK3R1*	chr5	67590448	T	G	1988	8.8	Y504D
8287	*PIK3R1*	chr5	67590448	T	G	1990	7.3	Y504D
8531	*PIK3R1*	chr5	67590448	T	G	1988	4.9	Y504D
8715	*PIK3R1*	chr5	67590448	T	G	1989	4	Y504D
8715	*PIK3R1*	chr5	67590448	T	G	1992	3.4	Y504D
4487	*PTEN*	chr10	89720696	C	G	90	4.4	P283A
6740	*PTEN*	chr10	89720696	C	G	81	8.6	P283A
6740	*PTEN*	chr10	89725149	A	G	997	3	R378G
9557	*PTEN*	chr10	89720696	C	G	130	5.4	P283A
9841	*PTEN*	chr10	89720696	C	G	155	3.9	P283A
9843	*PTEN*	chr10	89692792	C	G	1999	25.3	D92E
2226	*PTEN*	chr10	89720696	C	G	80	17.5	P283A
4377	*PTEN*	chr10	89725149	A	G	693	3	R378G
6705	*PTEN*	chr10	89720696	C	G	95	5.3	P283A
6768	*PTEN*	chr10	89720696	C	G	104	12.5	P283A
7717	*PTEN*	chr10	89720696	C	G	99	6.1	P283A
0585	*PTEN*	chr10	89720696	C	G	96	10.4	P283A
1590	*PTEN*	chr10	89720696	C	G	129	6.2	P283A
8720	*PTEN*	chr10	89692917	T	C	616	47.6	M134C
9238	*PTEN*	chr10	89692980	A	C	747	12.6	Y155S
9444	*PTEN*	chr10	89720696	C	G	70	7.1	P283A
9444	*PTEN*	chr10	89725149	A	G	905	3.1	R378G
2347	*PTEN*	chr10	89725051	T	G	318	50.3	L345R
0056	*PTEN*	chr10	89725111	T	A	127	5.5	V365E

Abbreviations: Chrom, chromosome; Ref, reference nucleotide; AA, Amino Acid.

Supplementary Table 3 Distribution of *PIK3CA* mutations in various functional domains

	TCGA[1]					FUSCC[2]				
domains	N	N/PCM	(%)	N/Total	(%)	N	N/PCM	(%)	N/Total	(%)
PI3K-ABD	5	5/317	2%	5/1105	0%	11	11/89	12%	11/149	7%
PI3K-RBD	0	0/317	0%	0/1105	0%	7	7/89	8%	7/149	5%
C2	35	35/317	11%	35/1105	3%	4	4/89	4%	4/149	3%
Helical	120	120/317	38%	120/1105	11%	13	13/89	15%	13/149	9%
PI4K	142	142/317	45%	142/1105	13%	39	39/89	44%	39/149	26%
Linker region	21	21/317	7%	21/1105	2%	15	15/89	17%	15/149	10%

Abbreviation: ABD, adaptor-binding domain; RBD, Ras-binding domain.

Note: Mutation denotes somatic missense mutation;

[1]TCGA: *PIK3CA* mutation (PCM), 317 cases; Total, 1105 cases;

[2]FUSCC: *PIK3CA* mutation (PCM), 89 cases; Total, 149 cases;

Supplementary Table 4 Distribution of *PIK3R1* mutations in various functional domains

	TCGA[1]					FUSCC[2]				
domains	N	N/PRM	(%)	N/Total	(%)	N	N/PRM	(%)	N/Total	(%)
SH3	0	0/7	0%	0/1105	0%	0	0/38	0%	0/149	0%
Rho-GAP	0	0/7	0%	0/1105	0%	5	5/38	13%	5/149	3%
nSH2	0	0/7	0%	0/1105	0%	0	0/38	0%	0/149	0%
iSH2	4	4/7	57%	4/1105	0%	15	15/38	39%	15/149	10%
cSH2	3	3/7	43%	3/1105	0%	7	7/38	18%	7/149	5%
Linker region	0	0/7	0%	0/1105	0%	11	11/38	29%	11/149	7%

Abbreviation: nSH2, N-terminal SH2 domain; iSH2, inter-SH2 domain; cSH2, C-terminal SH2 domain.

Note: Mutation denotes somatic missense mutation;

[1]TCGA: *PIK3R1* mutation (PRM), 7 cases; Total, 1105 cases;

[2]FUSCC: *PIK3R1* mutation (PRM), 38 cases; Total, 149 cases;

Supplementary Table 5 Avg ES of *PIK3CA* mutations in ReMB library sceening for cellular proliferation

DNA Change[a]	Protein[b]	Domain	Avg ES[c]（MCF-10A）	Avg ES[c]（HMEC）	Classification[d]
wild-type	/	/	-0.096	-0.187	
a3140t	p.H1047L	PI3K/PI4K	2.486	1.578	Y
g3145c	p.G1049R	PI3K/PI4K	2.203	0.883	Y
a3140g	p.H1047R	PI3K/PI4K	1.840	1.067	Y
g1357a	p.E453K	C2 PI3K-type	2.084	0.630	Y
a1034t	p.N345I	C2 PI3K-type	1.516	0.741	Y
g1624a	p.E542K	PIK helical	1.623	0.517	Y
g1633a	p.E545K	PIK helical	1.324	0.770	Y
g115a	p.E39K	PI3K-ABD	1.408	0.648	Y
c3139a_a3140c	p.H1047T	PI3K/PI4K	1.585	0.346	
a3127g	p.M1043V	PI3K/PI4K	1.646	0.280	
a3130t	p.N1044Y	PI3K/PI4K	1.157	0.753	
g2702t	p.C901F	PI3K/PI4K	1.218	0.633	
a1634c	p.E545A	PIK helical	1.349	0.476	
a1625t	p.E542V	PIK helical	1.292	0.430	
a2816g	p.D939G	PI3K/PI4K	1.381	0.315	
c311t	p.P104L	PI3K-ABD	1.210	0.433	
a1637t	p.Q546L	PIK helical	0.982	0.658	
a3194t	p.H1065L	PI3K/PI4K	0.981	0.659	
g241a	p.E81K	PI3K-ABD	0.994	0.635	
a1049g	p.D350G	C2 PI3K-type	1.175	0.435	
c3139t	p.H1047Y	PI3K/PI4K	1.099	0.509	
t1035g	p.N345K	C2 PI3K-type	1.236	0.354	
a1637c	p.Q546P	PIK helical	0.936	0.641	
c1636g	p.Q546E	PIK helical	1.068	0.490	
a1637g	p.Q546R	PIK helical	1.093	0.430	
t3132g	p.N1044K	PI3K/PI4K	0.967	0.491	
g3129a	p.M1043I	PI3K/PI4K	1.068	0.376	
c1886g	p.S629C	PIK helical	0.982	0.455	
c3104t	p.A1035V	PI3K/PI4K	1.125	0.310	
g1624c	p.E542Q	PIK helical	0.974	0.459	
a1634g	p.E545G	PIK helical	1.169	0.241	
p.E365K	p.E365K	C2 PI3K-type	1.119	0.282	
g2740a	p.G914R	PI3K/PI4K	0.901	0.471	
g3019c	p.G1007R	PI3K/PI4K	1.003	0.354	
g1252a	p.E418K	C2 PI3K-type	0.629	0.710	
a3062g	p.Y1021C	PI3K/PI4K	0.919	0.419	
c1618t	p.L540F	PIK helical	0.694	0.626	
a1033c	p.N345H	C2 PI3K-type	0.756	0.563	
c3074g	p.T1025S	PI3K/PI4K	0.696	0.614	
g2908a	p.E970K	PI3K/PI4K	0.857	0.446	
c2296t	p.L766F	NONE	0.933	0.271	
c3155a	p.T1052K	PI3K/PI4K	0.774	0.429	
g3012a	p.M1004I	PI3K/PI4K	0.771	0.377	
c1214t	p.S405F	C2 PI3K-type	0.869	0.264	
a3143t	p.H1048L	PI3K/PI4K	0.808	0.309	
g2176a	p.E726K	NONE	0.628	0.442	
c2155g	p.L719V	NONE	0.496	0.543	
g353a	p.G118D	NONE	0.876	0.161	
c2727a	p.F909L	PI3K/PI4K	0.689	0.337	
c1097g	p.P366R	C2 PI3K-type	0.746	0.244	
g3085c	p.D1029H	PI3K/PI4K	0.613	0.345	
g113a	p.R38H	PI3K-ABD	0.630	0.320	
g323a	p.R108H	NONE	0.920	0.019	
c1616g	p.P539R	PIK helical	0.634	0.275	
c1598t	p.A533V	PIK helical	0.539	0.368	
a1490g	p.N497S	NONE	0.504	0.398	
c1371g	p.N457K	C2 PI3K-type	0.542	0.337	
a2279t	p.Q760L	NONE	0.630	0.242	

a1094t	p.E365V	C2 PI3K-type	0.426	0.441
g238a	p.E80K	PI3K-ABD	0.492	0.341
c1697t	p.P566L	PIK helical	0.603	0.212
c3137t	p.A1046V	PI3K/PI4K	0.407	0.400
g3136a_a3138g	p.A1046T	PI3K/PI4K	0.307	0.460
a3073g	p.T1025A	PI3K/PI4K	0.495	0.266
a604g	p.N202D	PI3K-RBD	0.296	0.462
a3143g	p.H1048R	PI3K/PI4K	0.634	0.122
c3197t	p.A1066V	PI3K/PI4K	0.600	0.132
g3034a	p.E1012K	PI3K/PI4K	0.333	0.394
g1030a	p.V344M	C2 PI3K-type	0.524	0.184
g3146c	p.G1049A	PI3K/PI4K	0.576	0.130
g3109a	p.E1037K	PI3K/PI4K	0.529	0.167
g263a	p.R88Q	PI3K-ABD	0.794	-0.129
t2017a	p.S673T	PIK helical	0.455	0.198
c3074t	p.T1025I	PI3K/PI4K	0.630	-0.002
g2119a	p.E707K	NONE	0.274	0.354
t1568c	p.L523S	PIK helical	0.346	0.252
g328a	p.E110K	NONE	0.124	0.450
g1133t	p.C378F	C2 PI3K-type	0.500	0.071
a3118g	p.M1040V	PI3K/PI4K	0.159	0.402
t1395g	N465K	C2 PI3K-type	0.131	0.366
g1633c	p.E545Q	PIK helical	0.345	0.147
t632g	p.I211S	PI3K-RBD	0.354	0.122
g1193a	p.R398H	C2 PI3K-type	0.230	0.245
a1543g	p.N515D	NONE	0.182	0.279
g1639a	p.E547K	PIK helical	0.229	0.221
g3068t	p.R1023L	PI3K/PI4K	0.167	0.253
a1540g	p.S514G	NONE	0.483	-0.079
t1840a	p.F614I	PIK helical	0.223	0.181
c2965g	p.L989V	PI3K/PI4K	0.119	0.249
a3184g	p.I1062V	PI3K/PI4K	0.064	0.301
a3154g	p.T1052A	PI3K/PI4K	0.259	0.101
a1546g	p.R516G	PIK helical	0.166	0.132
a1173g	p.I391M	C2 PI3K-type	0.225	0.064
a2102c	p.H701P	NONE	0.020	0.177
g3120a	p.M1040I	PI3K/PI4K	-0.094	0.245
a3121g	p.K1041E	PI3K/PI4K	-0.192	0.214
c178a	p.Q60K	PI3K-ABD	-0.114	0.125
a93g	p.I31M	PI3K-ABD	-0.117	0.125
g1807c	p.D603H	PIK helical	0.052	-0.052
c3059t	p.A1020V	PI3K/PI4K	0.064	-0.073
a656g	p.Q219R	PI3K-RBD	0.047	-0.101
a2129g	p.E710G	NONE	-0.057	-0.002
g1612t	p.D538Y	PIK helical	-0.436	0.235
a153t	p.K51N	PI3K-ABD	-0.012	-0.232

[a]The GenBank accession number of transcript is NM_006218.2.

[b]The GenBank accession number of protein is NP_006209.2.

[c]Average enrichment score（Avg ES）indicates the average of two biological replicates.

[d]Proliferation-driven mutations were determined with the mean Avg ES above 1 in MCF-10A and HMEC cell lines.

Supplementary Table 6 Avg ES of *PIK3CA* mutations in ReMB library sceening for doxorubicin response

DNA Change[a]	Protein[b]	Domain	Avg ES[c]（MCF-10A）	Avg ES[c]（HMEC）	Classification[d]
wild-type	/	/	-0.051	-0.109	
a3140t	p.H1047L	PI3K/PI4K	1.811	2.223	Y
a3140g	p.H1047R	PI3K/PI4K	1.335	2.015	Y
g3145c	p.G1049R	PI3K/PI4K	1.577	1.711	Y
g1357a	p.E453K	C2 PI3K-type	1.520	1.335	Y
c3139a_a3140c	p.H1047T	PI3K/PI4K	1.192	1.584	Y
a3127g	p.M1043V	PI3K/PI4K	1.107	1.200	Y
a1034t	p.N345I	C2 PI3K-type	1.117	1.101	Y
t1035g	p.N345K	C2 PI3K-type	0.913	1.252	Y
g1624a	p.E542K	PIK helical	1.068	1.087	Y
g115a	p.E39K	PI3K-ABD	1.021	0.948	
a1637g	p.Q546R	PIK helical	0.757	1.186	
a1637c	p.Q546P	PIK helical	0.907	0.965	
a2816g	p.D939G	PI3K/PI4K	1.077	0.768	
a1625t	p.E542V	PIK helical	0.814	1.020	
a1634c	p.E545A	PIK helical	1.049	0.737	
g3129a	p.M1043I	PI3K/PI4K	0.836	0.905	
g1624c	p.E542Q	PIK helical	0.688	0.988	
a3130t	p.N1044Y	PI3K/PI4K	0.720	0.947	
g1633a	p.E545K	PIK helical	0.868	0.785	
g2702t	p.C901F	PI3K/PI4K	0.730	0.862	
t3132g	p.N1044K	PI3K/PI4K	0.629	0.892	
c3104t	p.A1035V	PI3K/PI4K	0.715	0.802	
p.E365K	p.E365K	C2 PI3K-type	0.803	0.706	
a1634g	p.E545G	PIK helical	0.783	0.724	
a1049g	p.D350G	C2 PI3K-type	0.567	0.917	
c3139t	p.H1047Y	PI3K/PI4K	0.634	0.799	
c311t	p.P104L	PI3K-ABD	0.788	0.602	
a1637t	p.Q546L	PIK helical	0.752	0.612	
c1636g	p.Q546E	PIK helical	0.589	0.699	
g2908a	p.E970K	PI3K/PI4K	0.613	0.621	
a3062g	p.Y1021C	PI3K/PI4K	0.809	0.416	
g241a	p.E81K	PI3K-ABD	0.533	0.671	
g353a	p.G118D	NONE	0.562	0.600	
c2296t	p.L766F	NONE	0.753	0.400	
c1886g	p.S629C	PIK helical	0.655	0.475	
g3019c	p.G1007R	PI3K/PI4K	0.511	0.604	
a3194t	p.H1065L	PI3K/PI4K	0.408	0.704	
c1214t	p.S405F	C2 PI3K-type	0.489	0.609	
a3143t	p.H1048L	PI3K/PI4K	0.571	0.498	
c3155a	p.T1052K	PI3K/PI4K	0.479	0.524	
c2727a	p.F909L	PI3K/PI4K	0.453	0.526	
g323a	p.R108H	NONE	0.572	0.397	
c1097g	p.P366R	C2 PI3K-type	0.242	0.685	
a3143g	p.H1048R	PI3K/PI4K	0.404	0.516	
g3146c	p.G1049A	PI3K/PI4K	0.300	0.592	
g2176a	p.E726K	NONE	0.222	0.634	
g1252a	p.E418K	C2 PI3K-type	0.305	0.448	
g113a	p.R38H	PI3K-ABD	0.295	0.450	
g2740a	p.G914R	PI3K/PI4K	0.450	0.286	
c1616g	p.P539R	PIK helical	0.149	0.539	
c1618t	p.L540F	PIK helical	0.182	0.500	
c3074g	p.T1025S	PI3K/PI4K	0.224	0.454	
g3012a	p.M1004I	PI3K/PI4K	0.403	0.272	
g1030a	p.V344M	C2 PI3K-type	0.229	0.426	
c3074t	p.T1025I	PI3K/PI4K	0.193	0.432	
g263a	p.R88Q	PI3K-ABD	0.217	0.400	
a3073g	p.T1025A	PI3K/PI4K	0.370	0.237	
c1598t	p.A533V	PIK helical	0.212	0.390	

t632g	p.I211S	PI3K-RBD	0.256	0.296
a1033c	p.N345H	C2 PI3K-type	0.381	0.156
g238a	p.E80K	PI3K-ABD	0.286	0.242
g3085c	p.D1029H	PI3K/PI4K	0.199	0.319
g3109a	p.E1037K	PI3K/PI4K	0.266	0.190
c3197t	p.A1066V	PI3K/PI4K	0.167	0.207
a2279t	p.Q760L	NONE	0.159	0.108
t1568c	p.L523S	PIK helical	0.182	0.078
c1697t	p.P566L	PIK helical	0.196	0.056
a1490g	p.N497S	NONE	-0.008	0.245
g1133t	p.C378F	C2 PI3K-type	0.124	0.099
a1546g	p.R516G	PIK helical	-0.035	0.251
c2155g	p.L719V	NONE	0.078	0.105
c1371g	p.N457K	C2 PI3K-type	0.247	-0.077
g1633c	p.E545Q	PIK helical	-0.002	0.156
a1543g	p.N515D	NONE	0.010	0.106
a3154g	p.T1052A	PI3K/PI4K	0.104	-0.046
c3137t	p.A1046V	PI3K/PI4K	0.087	-0.046
a1540g	p.S514G	NONE	0.225	-0.201
g3034a	p.E1012K	PI3K/PI4K	-0.113	0.136
g3136a_a3138g	p.A1046T	PI3K/PI4K	0.098	-0.105
t2017a	p.S673T	PIK helical	0.246	-0.271
g3068t	p.R1023L	PI3K/PI4K	0.023	-0.068
a3118g	p.M1040V	PI3K/PI4K	-0.246	0.191
a1173g	p.I391M	C2 PI3K-type	-0.004	-0.080
a1094t	p.E365V	C2 PI3K-type	0.059	-0.216
g1193a	p.R398H	C2 PI3K-type	-0.051	-0.106
c2965g	p.L989V	PI3K/PI4K	0.009	-0.192
a3184g	p.I1062V	PI3K/PI4K	-0.054	-0.138
t1395g	N465K	C2 PI3K-type	-0.156	-0.037
t1840a	p.F614I	PIK helical	-0.080	-0.117
a604g	p.N202D	PI3K-RBD	-0.090	-0.254
c3059t	p.A1020V	PI3K/PI4K	-0.146	-0.225
a2102c	p.H701P	NONE	-0.056	-0.336
g1639a	p.E547K	PIK helical	-0.092	-0.310
g3120a	p.M1040I	PI3K/PI4K	-0.329	-0.076
g2119a	p.E707K	NONE	-0.139	-0.297
g328a	p.E110K	NONE	-0.236	-0.206
a656g	p.Q219R	PI3K-RBD	-0.305	-0.143
a153t	p.K51N	PI3K-ABD	-0.314	-0.238
g1807c	p.D603H	PIK helical	-0.309	-0.270
g1612t	p.D538Y	PIK helical	-0.549	-0.076
c178a	p.Q60K	PI3K-ABD	-0.426	-0.374
a3121g	p.K1041E	PI3K/PI4K	-0.401	-0.472
a2129g	p.E710G	NONE	-0.098	-0.818
a93g	p.I31M	PI3K-ABD	-0.473	-0.472

[a]The GenBank accession number of transcript is NM_006218.2.

[b]The GenBank accession number of protein is NP_006209.2.

[c]Average enrichment score（Avg ES）indicates the average of two biological replicates.

[d]Doxorubicin resistant mutations were determined with the mean Avg ES above 1 in MCF-10A and HMEC cell lines.

Supplementary Table 7 Avg ES of *PIK3CA* mutations in ReMB library sceening for BKM120 response

DNA Change[a]	Protein[b]	Domain	Avg ES[c] （MCF-10A）	Avg ES[c] （HMEC）	Classification[d]
wild-type	/	/	0.090	0.146	
a3140t	p.H1047L	PI3K/PI4K	2.431	1.643	Y
g3145c	p.G1049R	PI3K/PI4K	2.148	0.949	Y
a3140g	p.H1047R	PI3K/PI4K	1.785	1.133	Y
g1357a	p.E453K	C2 PI3K-type	2.029	0.696	Y
a1034t	p.N345I	C2 PI3K-type	1.461	0.807	Y
g1624a	p.E542K	PIK helical	1.568	0.583	Y
g1633a	p.E545K	PIK helical	1.269	0.836	Y
g115a	p.E39K	PI3K-ABD	1.354	0.713	Y
t1035g	p.N345K	C2 PI3K-type	0.989	0.985	
c3139a_a3140c	p.H1047T	PI3K/PI4K	0.945	1.029	
a1637g	p.Q546R	PIK helical	0.846	1.120	
a3127g	p.M1043V	PI3K/PI4K	1.053	0.906	
a2816g	p.D939G	PI3K/PI4K	1.157	0.784	
a1625t	p.E542V	PIK helical	0.886	1.018	
a1634c	p.E545A	PIK helical	1.050	0.762	
g2702t	p.C901F	PI3K/PI4K	0.963	0.822	
t3132g	p.N1044K	PI3K/PI4K	0.816	0.926	
a3130t	p.N1044Y	PI3K/PI4K	0.759	0.954	
a1637c	p.Q546P	PIK helical	0.690	1.016	
g1624c	p.E542Q	PIK helical	0.703	0.990	
p.E365K	p.E365K	C2 PI3K-type	0.962	0.681	
c3139t	p.H1047Y	PI3K/PI4K	0.796	0.837	
a1049g	p.D350G	C2 PI3K-type	0.630	0.998	
a1634g	p.E545G	PIK helical	0.770	0.709	
c311t	p.P104L	PI3K-ABD	0.849	0.607	
c3104t	p.A1035V	PI3K/PI4K	0.643	0.789	
g3129a	p.M1043I	PI3K/PI4K	0.618	0.757	
c1636g	p.Q546E	PIK helical	0.647	0.708	
a1637t	p.Q546L	PIK helical	0.624	0.716	
g241a	p.E81K	PI3K-ABD	0.560	0.702	
a3194t	p.H1065L	PI3K/PI4K	0.562	0.660	
g3019c	p.G1007R	PI3K/PI4K	0.581	0.606	
c1214t	p.S405F	C2 PI3K-type	0.610	0.572	
g2908a	p.E970K	PI3K/PI4K	0.549	0.631	
c1886g	p.S629C	PIK helical	0.748	0.426	
c1097g	p.P366R	C2 PI3K-type	0.395	0.689	
c2727a	p.F909L	PI3K/PI4K	0.592	0.485	
c2296t	p.L766F	NONE	0.660	0.414	
a3062g	p.Y1021C	PI3K/PI4K	0.630	0.414	
g353a	p.G118D	NONE	0.422	0.534	
g3146c	p.G1049A	PI3K/PI4K	0.420	0.534	
g2740a	p.G914R	PI3K/PI4K	0.620	0.333	
a3143t	p.H1048L	PI3K/PI4K	0.407	0.513	
g323a	p.R108H	NONE	0.578	0.328	
c3155a	p.T1052K	PI3K/PI4K	0.392	0.491	
c3074t	p.T1025I	PI3K/PI4K	0.435	0.401	
c1618t	p.L540F	PIK helical	0.311	0.503	
g2176a	p.E726K	NONE	0.267	0.533	
a3143g	p.H1048R	PI3K/PI4K	0.246	0.508	
c3074g	p.T1025S	PI3K/PI4K	0.259	0.471	
g1252a	p.E418K	C2 PI3K-type	0.227	0.497	
c1616g	p.P539R	PIK helical	0.213	0.510	
g263a	p.R88Q	PI3K-ABD	0.243	0.405	
g3085c	p.D1029H	PI3K/PI4K	0.286	0.361	
g3012a	p.M1004I	PI3K/PI4K	0.338	0.249	
a3073g	p.T1025A	PI3K/PI4K	0.354	0.229	
a1033c	p.N345H	C2 PI3K-type	0.460	0.101	
g113a	p.R38H	PI3K-ABD	0.180	0.373	
g1030a	p.V344M	C2 PI3K-type	0.109	0.427	
c1598t	p.A533V	PIK helical	0.120	0.377	
t632g	p.I211S	PI3K-RBD	0.160	0.292	

c3197t	p.A1066V	PI3K/PI4K	0.154	0.220
g238a	p.E80K	PI3K-ABD	0.187	0.183
a1490g	p.N497S	NONE	0.053	0.260
g3109a	p.E1037K	PI3K/PI4K	0.172	0.124
a2279t	p.Q760L	NONE	0.243	0.053
c1371g	p.N457K	C2 PI3K-type	0.246	-0.045
a1546g	p.R516G	PIK helical	-0.014	0.200
g1133t	p.C378F	C2 PI3K-type	0.062	0.108
g3034a	p.E1012K	PI3K/PI4K	-0.032	0.175
a1543g	p.N515D	NONE	-0.023	0.160
c1697t	p.P566L	PIK helical	0.105	0.026
t1568c	p.L523S	PIK helical	0.107	0.017
c2155g	p.L719V	NONE	-0.066	0.171
g1633c	p.E545Q	PIK helical	-0.107	0.189
a1540g	p.S514G	NONE	0.141	-0.199
c3137t	p.A1046V	PI3K/PI4K	-0.043	-0.015
a1173g	p.I391M	C2 PI3K-type	-0.017	-0.050
t2017a	p.S673T	PIK helical	-0.008	-0.063
a3184g	p.I1062V	PI3K/PI4K	0.037	-0.116
a3154g	p.T1052A	PI3K/PI4K	-0.166	-0.017
g3136a_a3138g	p.A1046T	PI3K/PI4K	-0.091	-0.120
a1094t	p.E365V	C2 PI3K-type	-0.035	-0.197
g3068t	p.R1023L	PI3K/PI4K	-0.141	-0.106
a656g	p.Q219R	PI3K-RBD	-0.146	-0.105
g2119a	p.E707K	NONE	-0.136	-0.139
a3118g	p.M1040V	PI3K/PI4K	-0.413	0.136
t1395g	N465K	C2 PI3K-type	-0.235	-0.090
g1193a	p.R398H	C2 PI3K-type	-0.263	-0.072
c2965g	p.L989V	PI3K/PI4K	-0.137	-0.207
g328a	p.E110K	NONE	-0.052	-0.308
t1840a	p.F614I	PIK helical	-0.298	-0.093
c3059t	p.A1020V	PI3K/PI4K	-0.183	-0.268
g3120a	p.M1040I	PI3K/PI4K	-0.395	-0.134
g1639a	p.E547K	PIK helical	-0.289	-0.249
a2102c	p.H701P	NONE	-0.279	-0.338
g1807c	p.D603H	PIK helical	-0.438	-0.189
a604g	p.N202D	PI3K-RBD	-0.409	-0.268
g1612t	p.D538Y	PIK helical	-0.687	-0.013
c178a	p.Q60K	PI3K-ABD	-0.524	-0.264
a153t	p.K51N	PI3K-ABD	-0.442	-0.354
a93g	p.I31M	PI3K-ABD	-0.529	-0.431
a3121g	p.K1041E	PI3K/PI4K	-0.628	-0.525
a2129g	p.E710G	NONE	-0.466	-0.740

[a]The GenBank accession number of transcript is NM_006218.2.

[b]The GenBank accession number of protein is NP_006209.2.

[c]Average enrichment score（Avg ES）indicates the average of two biological replicates.

[d]BKM120 resistant mutations were determined with the mean Avg ES above 1 in MCF-10A and HMEC cell lines.

Supplementary Table 8 Avg ES of *PIK3R1* mutations in ReMB library sceening for cellular proliferation

DNA Change[a]	Protein[b]	Domain	Avg ES[c]（MCF-10A）	Avg ES[c]（HMEC）	Classification[d]
wild-type	/	/	-0.240	-0.288	
a480c	p.E160D	Rho-GAP	1.451	1.432	Y
a1690g	p.N564D	NONE	1.344	0.954	Y
a2021g	p.K674R	SH2-2	1.246	1.043	Y
a986t	p.Q329L	NONE	0.819	0.782	Y
g1678t	p.D560Y	NONE	0.890	0.722	Y
g1721c	p.R574T	NONE	0.860	0.591	
a1895t	p.N623I	SH2-2	0.749	0.566	
g978a	p.M326I	NONE	0.603	0.512	
g1390a	p.D464N	NONE	0.629	0.417	
g1543a	p.E515K	NONE	0.566	0.658	
g1903a	p.E635K	SH2-2	0.704	0.482	
a1529t	p.E510V	NONE	0.618	0.446	
c251t	p.P84L	NONE	0.512	0.558	
t1676c	p.I559T	NONE	0.521	0.386	
g532a	p.D178N	Rho-GAP	0.483	0.389	
c1785g	p.N595K	NONE	0.417	0.523	
a2063c	p.Y688S	SH2-2	0.442	0.433	
t1510g	p.Y504D	NONE	0.301	0.435	
a38t	p.D13V	SH3	0.379	0.332	
a2018c	p.N673T	SH2-2	0.323	0.209	
g1900c	p.A634P	SH2-2	0.189	0.204	
t1346c	p.L449S	NONE	-0.264	0.098	
g1925t	p.R642L	SH2-2	-0.327	0.144	
g1930a	p.G644S	SH2-2	-0.237	-0.023	
t1676a	p.I559N	NONE	-0.230	-0.011	
a1319g	p.D440G	NONE	-0.182	0.152	
g1930t	p.G644C	SH2-2	-0.217	0.024	

[a]The GenBank accession number of transcript is NM_181523.2.
[b]The GenBank accession number of protein is NP_852664.1.
[c]Average enrichment score（Avg ES）indicates the average of two biological replicates.
[d]Proliferation-driven mutations were determined with the mean Avg ES above 0.8 in MCF-10A and HMEC cell lines.

Supplementary Table 9 Avg ES of *PIK3R1* mutations in ReMB library sceening for doxorubicin response

DNA Change[a]	Protein[b]	Domain	Avg ES[c]（MCF-10A）	Avg ES[c]（HMEC）	Classification[d]
wild-type	/	/	-0.290	-0.331	
g1678t	p.D560Y	NONE	1.833	1.846	Y
g1721c	p.R574T	NONE	1.428	1.835	Y
a1690g	p.N564D	NONE	1.481	1.132	Y
a480c	p.E160D	Rho-GAP	1.159	1.051	Y
a2021g	p.K674R	SH2-2	1.030	1.052	Y
a986t	p.Q329L	NONE	0.892	0.860	Y
g1543a	p.E515K	NONE	0.793	0.783	
a1895t	p.N623I	SH2-2	0.722	0.806	
g978a	p.M326I	NONE	0.591	0.725	
g1390a	p.D464N	NONE	0.566	0.658	
g1903a	p.E635K	SH2-2	0.512	0.558	
a1529t	p.E510V	NONE	0.417	0.523	
t1676c	p.I559T	NONE	0.482	0.593	
g532a	p.D178N	Rho-GAP	0.446	0.532	
a2063c	p.Y688S	SH2-2	0.386	0.453	
t1510g	p.Y504D	NONE	0.389	0.436	
t1346c	p.L449S	NONE	0.433	0.437	
g1930a	p.G644S	SH2-2	0.435	0.368	
g1900c	p.A634P	SH2-2	0.332	0.356	
c251t	p.P84L	NONE	0.209	0.266	
g1930t	p.G644C	SH2-2	0.204	0.197	
g1925t	p.R642L	SH2-2	0.098	-0.083	
a38t	p.D13V	SH3	0.144	-0.091	
t1676a	p.I559N	NONE	-0.023	-0.130	
a1319g	p.D440G	NONE	-0.011	-0.120	
a2018c	p.N673T	SH2-2	0.152	-0.015	
c1785g	p.N595K	NONE	0.024	-0.096	

[a]The GenBank accession number of transcript is NM_181523.2.
[b]The GenBank accession number of protein is NP_852664.1.
[c]Average enrichment score（Avg ES）indicates the average of two biological replicates.
[d]Doxorubicin resistant mutations were determined with the mean Avg ES above 0.8 in MCF-10A and HMEC cell lines.

Supplementary Table 10 Avg ES of *PIK3R1* mutations in ReMB library sceening for BKM120 response

DNA Change[a]	Protein[b]	Domain	Avg ES[c]（MCF-10A）	Avg ES[c]（HMEC）	Classification[d]
wild-type	/	/	-0.29043	-0.3309725	
g1678t	p.D560Y	NONE	1.533	1.546	Y
a1690g	p.N564D	NONE	1.128	1.535	Y
g1721c	p.R574T	NONE	1.181	0.832	Y
a480c	p.E160D	Rho-GAP	0.796	0.921	Y
a986t	p.Q329L	NONE	0.859	0.751	Y
c1785g	p.N595K	NONE	0.730	0.752	
a2021g	p.K674R	SH2-2	0.692	0.710	
g1543a	p.E515K	NONE	0.629	0.692	
a1895t	p.N623I	SH2-2	0.481	0.637	
g1903a	p.E635K	SH2-2	0.553	0.654	
g1390a	p.D464N	NONE	0.470	0.697	
t1676c	p.I559T	NONE	0.400	0.570	
g532a	p.D178N	Rho-GAP	0.388	0.551	
a1529t	p.E510V	NONE	0.473	0.517	
a2018c	p.N673T	SH2-2	0.252	0.632	
a2063c	p.Y688S	SH2-2	0.333	0.465	
t1510g	p.Y504D	NONE	0.426	0.353	
a38t	p.D13V	SH3	0.219	-0.148	
g978a	p.M326I	NONE	0.108	0.321	
g1925t	p.R642L	SH2-2	0.100	0.325	
g1900c	p.A634P	SH2-2	0.011	0.179	
t1346c	p.L449S	NONE	-0.700	-0.555	
g1930a	p.G644S	SH2-2	-0.577	-0.605	
t1676a	p.I559N	NONE	-0.505	-0.546	
c251t	p.P84L	NONE	-0.355	-0.621	
a1319g	p.D440G	NONE	-0.466	-0.505	
g1930t	p.G644C	SH2-2	-0.447	-0.512	

[a]The GenBank accession number of transcript is NM_181523.2.
[b]The GenBank accession number of protein is NP_852664.1.
[c]Average enrichment score（Avg ES）indicates the average of two biological replicates.
[d]BKM120 resistant mutations were determined with the mean Avg ES above 0.8 in MCF-10A and HMEC cell lines.

Supplementary Table 11 Distribution of impactful *PIK3CA* mutations in various functional domains

		TCGA[1]					FUSCC[2]				
domains	Impactful *PIK3CA* mutation	N	N/IPCM	(%)	N/Total	(%)	N	N/IPCM	(%)	N/Total	(%)
PI3K-ABD	*E39K*	0	0/269	0%	0/1105	0%	1	1/49	1%	1/149	1%
PI3K-RBD		0	0/269	0%	0/1105	0%	0	0/49	0%	0/149	0%
C2	*N345K/I, E453K*	23	23/269	9%	35/1105	2%	4	4/49	4%	4/149	3%
Helical	*E542K, E545K*	104	104/269	39%	120/1105	9%	10	10/49	11%	10/149	7%
PI4K	*M1043V, H1047R/T/L, G1049R*	142	142/269	53%	143/1105	13%	34	34/49	38%	34/149	23%
Linker region		0	0/269	0%	0/1105	0%	0	0/49	0%	0/149	0%

Abbreviation: ABD, adaptor-binding domain; RBD, Ras-binding domain.

Note: Mutation denotes somatic missense mutation;

[1]TCGA: Impactful *PIK3CA* mutation (IPCM), 269 cases; Total, 1105 cases;

[2]FUSCC: Impactful *PIK3CA* mutation (IPCM), 49 cases; Total, 149 cases;

Supplementary Table 12 Distribution of impactful *PIK3R1* mutations in various functional domains

		TCGA[1]					FUSCC[2]				
domains	Impactful *PIK3R1* mutation	N	N/IPRM	(%)	N/Total	(%)	N	N/IPRM	(%)	N/Total	(%)
SH3		0	0/0	0%	0/1105	0%	0	0/8	0%	0/149	0%
Rho-GAP	*E160D*	0	0/0	0%	0/1105	0%	4	4/8	50%	4/149	3%
nSH2		0	0/0	0%	0/1105	0%	0	0/8	0%	0/149	0%
iSH2	*N564D*	0	0/0	0%	0/1105	0%	1	1/8	13%	1/149	1%
cSH2		0	0/0	0%	0/1105	0%	0	0/8	0%	0/149	0%
Linker region	*Q329L, K674R*	0	0/0	0%	0/1105	0%	3	3/8	38%	3/149	2%

Abbreviation: nSH2, N-terminal SH2 domain; iSH2, inter-SH2 domain; cSH2, C-terminal SH2 domain.

Note: Mutation denotes somatic missense mutation;

[1]TCGA: Impactful *PIK3R1* mutation (IPRM), 0 cases; Total, 1105 cases;

[2]FUSCC: Impactful *PIK3R1* mutation (IPRM), 8 cases; Total, 149 cases;

Supplementary Table 13 Clinicopathological variables and the mutation status of *PI3KCA* in 149 patients with breast cancer

Variables	Number of patients(%)	*PIK3CA* mutation		*P* value	*PIK3CA* impactful mutation			*P1*	*P*2
		Neg (%)	Pos (%)		No mutation	Neg (%)	Pos (%)		
Total	149	84(56.4)	65(43.6)		84(56.4)	20(13.4)	45(30.2)		
Age (median 51, range 25-72)				0.463				0.281	0.762
≤50 years	83(55.7)	49(32.9)	34(22.8)		49(32.9)	9(6.0)	25(16.8)		
>50years	66(44.3)	35(23.5)	31(20.8)		35(23.5)	11(7.4)	20(13.4)		
Menopausal status				0.884				0.874	0.776
Premenopause	93(62.4)	52(34.9)	41(27.5)		52(34.9)	12(8.0)	29(19.5)		
Postmenopause	56(37.6)	32(21.5)	24(16.1)		32(21.5)	8(5.4)	16(10.7)		
Tumor size				0.081				0.676	0.113
≤2cm	79(53.0)	49(32.9)	30(20.1)		49(32.9)	10(6.7)	20(13.4)		
>2, 5≤cm	66(44.3)	34(22.8)	32(21.5)		34(22.8)	10(6.7)	22(14.8)		
>5cm	4(2.7)	1(0.7)	3(2.0)		1(0.7)	0(0)	3(2.1)		
LN status				0.845				0.553	0.916
Negative	72(48.3)	40(26.8)	32(21.5)		40(26.8)	11(7.4)	21(14.1)		
Positive	77(51.7)	44(29.5)	33(22.1)		44(29.5)	9(6.0)	24(16.1)		
Grade									
1	9(6.0)	3(2.0)	6(4.0)	0.308	3(2.0)	2(1.3)	4(2.7)	0.449	0.401
2	82(55.1)	49(32.9)	33(22.2)		49(32.9)	10(6.7)	23(15.4)		
3	58(38.9)	32(21.5)	26(17.4)		32(21.5)	8(5.4)	18(12.1)		
ER status				0.060				0.362	0.064
Negative	44(29.5)	30(20.1)	14(9.4)		30(20.1)	5(3.4)	9(6.0)		
Positive	105(70.5)	54(36.3)	51(34.2)		54(36.3)	15(10.0)	36(24.2)		
PR status				0.464				0.570	0.192
Negative	53(35.6)	32(21.5)	21(14.1)		32(21.5)	9(6.0)	12(8.1)		
Positive	96(64.4)	52(34.9)	44(29.5)		52(34.9)	11(7.4)	33(22.1)		
HER-2/neu status				0.730				0.441	1.000
Negative	94(63.1)	54(36.2)	40(26.8)		54(36.2)	11(7.4)	29(19.5)		
Positive	55(36.9)	30(20.1)	25(16.8)		30(20.1)	9(6.0)	16(10.7)		
LVI				0.477				0.603	0.219
Negative	76(51.0)	45(30.2)	31(20.8)		45(30.2)	12(8.0)	19(12.8)		
Positive	73(49.0)	39(26.2)	34(22.8)		39(26.2)	8(5.4)	26(17.4)		
Chemotherapy				0.920				0.867	0.346
Doxorubicin based[a]	44(29.5)	25(16.8)	19(12.8)		25(16.8)	5(3.4)	14(9.4)		
Taxanes added[b]	93(62.4)	53(35.6)	40(26.8)		53(35.6)	14(9.4)	26(17.4)		
Capecitabine	6(4.0%)	2(1.3)	4(2.7)		2(1.3)	0(0)	4(2.7)		
None	6(4.0%)	4(2.7)	2(1.3)		4(2.7)	1(0.7)	1(0.7)		

Abbreviations: ER, estrogen receptor; HER-2, human epidermal growth factor receptor 2; PR, progesterone receptor; Neg, non-impactful mutation; Pos, impactful mutation; LN, lymph node; LVI,lymphatic vessel invasion; a: Anthracycline plus cyclophosphamide and 5-fluorouracil or anthracycline plus cyclophosphamide; b: Anthracycline-based chemotherapy followed by or combined with taxanes.

Note: Based on Pearson $\chi 2$ test, for which P is based on Fisher's exact test; P1, no mutation versus non-impactful mutation; P2, no mutation versus impactful mutation.

Supplementary Table 14 Clinicopathological variables and the impactful mutation status of domains in *PI3KCA* in 149 patients with breast cancer

Variables	Number of patients(%)	*PIK3CA* domain mutation									
		Neg (%)	ABD (%)	RBD (%)	C2 (%)	Helica (%)	PI4K (%)	Linker region (%)	*P1*	*P2*	*P3*
Total	149	104(69.8)	1(0.7)	0(0)	2(1.3)	10(6.7)	32(21.5)	0(0)			
Age (median 51, range 25-72)									0.872	0.339	**0.007**
≤50 years	83(55.7)	58(38.9)	1(0.7)	0(0)	1(0.7)	4(2.7)	19(12.8)	0(0)			
＞50years	66(44.3)	46(30.9)	0(0)	0(0)	1(0.7)	6(4.0)	13(8.7)	0(0)			
Menopausal status						`			0.740	0.476	0.460
Premenopause	93(62.4)	64(43.0)	1(0.7)	0(0)	1(0.7)	5(3.4)	22(14.8)	0(0)			
Postmenopause	56(37.6)	40(26.8)	0(0)	0(0)	1(0.7)	5(3.4)	10(6.7)	0(0)			
Tumor size									0.960	0.226	0.140
≤2cm	79(53.0)	59(39.6)	0(0)	0(0)	1(0.7)	3(2.0)	16(10.7)	0(0)			
＞2, 5≤cm	66(44.3)	44(29.5)	1(0.7)	0(0)	1(0.7)	7(4.7)	13(8.7)	0(0)			
＞5cm	4(2.7)	1(0.7)	0(0)	0(0)	0(0)	0(0)	3(2.0)	0(0)			
LN status									0.169	0.508	0.830
Negative	72(48.3)	51(34.2)	0(0)	0(0)	0(0)	6(4.0)	15(10.1)	0(0)			
Positive	77(51.7)	53(35.6)	1(0.7)	0(0)	2(1.3)	4(2.7)	17(11.4)	0(0)			
Grade									0.914	0.761	0.578
1	9(6.0)	5(3.4)	0(0)	0(0)	0(0)	1(0.7)	3(2.0)	0(0)			
2	82(55.1)	59(39.6)	1(0.7)	0(0)	1(0.7)	5(3.4)	16(10.7)	0(0)			
3	58(38.9)	40(26.8)	0(0)	0(0)	1(0.7)	4(2.7)	13(8.7)	0(0)			
ER status									0.316	0.124	0.207
Negative	44(29.5)	35(23.5)	1(0.7)	0(0)	0(0)	1(0.7)	7(4.7)	0(0)			
Positive	105(70.4)	69(46.3)	0(0)	0(0)	2(1.3)	9(6.0)	25(16.8)	0(0)			
PR status									0.257	0.065	0.069
Negative	53(35.6)	41(27.5)	1(0.7)	0(0)	0(0)	1(0.7)	10(6.7)	0(0)			
Positive	96(64.4)	63(42.3)	0(0)	0(0)	2(1.3)	9(6.0)	22(14.8)	0(0)			
HER-2/neu status									0.276	0.638	1.000
Negative	94(63.1)	65(43.6)	0(0)	0(0)	2(1.3)	7(4.7)	20(13.4)	0(0)			
Positive	55(36.9)	39(26.2)	1(0.7)	0(0)	0(0)	3(2.0)	12(8.1)	0(0)			
LVI									0.124	0.753	0.160
Negative	76(51.0)	57(38.2)	0(0)	0(0)	0(0)	6(4.0)	13(8.7)	0(0)			
Positive	73(49.0)	47(31.5)	1(0.7)	0(0)	2(1.3)	4(2.7)	19(12.8)	0(0)			
Chemotherapy									**0.001**	0.868	0.244
Doxorubicin based[a]	44(29.5)	30(20.1)	0(0)	0(0)	1(0.7)	3(2.0)	10(6.7)	0(0)			
Taxanes added[b]	93(62.4)	67(45.0)	1(0.7)	0(0)	0(0)	7(4.7)	18(12.1)	0(0)			
Capecitabine	6(4.0%)	2(1.3)	0(0)	0(0)	1(0.7)	0(0)	3(2.0)	0(0)			
None	6(4.0%)	5(3.4)	0(0)	0(0)	0(0)	0(0)	1(0.7)	0(0)			

Abbreviations: ER, estrogen receptor; HER-2, human epidermal growth factor receptor 2; PR, progesterone receptor; Neg, no mutation or non-impactful mutation;LN, lymph node; LVI,lymphatic vessel invasion. a: Anthracycline plus cyclophosphamide and 5-fluorouracil or anthracycline plus cyclophosphamide; b: Anthracycline-based chemotherapy followed by or combined with taxanes.

Note: Based on Pearson $\chi 2$ test, for which P is based on Fisher's exact test; Bold values denote *P* value < 0.05. P1, no mutation versus impactful mutation in C2 domain; P2, no mutation versus impactful mutation in Helica domain; P3, no mutation versus impactful mutation in PI4K kinase domain.
