# Supplementary material for: Characterization of PIK3CA and PIK3R1 somatic mutations in Chinese breast cancer patients
Source: Nat Commun. 2018 Apr 10;9:1357. doi: 10.1038/s41467-018-03867-9 (PMC5893593; doi:10.1038/s41467-018-03867-9)
Supplement: Supplementary file 3 — Description of Additional Supplementary Files [file 41467_2018_3867_MOESM3_ESM.pdf]

Description of Additional Supplementary Files

File Name: Supplementary Data 1
Description: The spread sheet of initial mutations calling for breast tumors from the Ion Amplicon sequencing using Torrent Suite Software v3.2.
